# The impact of rearing environment on the development of gut microbiota in tilapia larvae

**DOI:** 10.1038/srep18206

**Published:** 2015-12-11

**Authors:** Christos Giatsis, Detmer Sipkema, Hauke Smidt, Hans Heilig, Giulia Benvenuti, Johan Verreth, Marc Verdegem

**Affiliations:** 1 Aquaculture and Fisheries Group, Wageningen University, PO Box 338, 6708 WD Wageningen, the Netherlands; 2 Laboratory of Microbiology, Wageningen University, Dreijenplein 10, 6703 HB Wageningen, the Netherlands; 3 Bioprocess Engineering, AlgaePARC, Wageningen University, PO Box 16, 6700 AA Wageningen, the Netherlands

## Abstract

This study explores the effect of rearing environment on water bacterial communities (BC) and the association with those present in the gut of Nile tilapia larvae (*Oreochromis niloticus*, Linnaeus) grown in either recirculating or active suspension systems. 454 pyrosequencing of PCR-amplified 16S rRNA gene fragments was applied to characterize the composition of water, feed and gut bacteria communities. Observed changes in water BC over time and differences in water BCs between systems were highly correlated with corresponding water physico-chemical properties. Differences in gut bacterial communities during larval development were correlated with differences in water communities between systems. The correlation of feed BC with those in the gut was minor compared to that between gut and water, reflected by the fact that 4 to 43 times more OTUs were shared between water and gut than between gut and feed BC. Shared OTUs between water and gut suggest a successful transfer of microorganisms from water into the gut, and give insight about the niche and ecological adaptability of water microorganisms inside the gut. These findings suggest that steering of gut microbial communities could be possible through water microbial management derived by the design and functionality of the rearing system.

Gut microbiota influences a wide range of biological processes of their host, including digestion, innate immunity, proliferation of epithelial cells and structural and functional maturation of the gut in humans^1^,^2^, domesticated terrestrial animals^3^,^4^ and fish^5^,^6^. However, our understanding of the roles and drivers for community diversity and species dominance within the gut is presently limited^7^.

Fish are exposed to higher microbial loads in the aquatic environment than terrestrial domesticated animals are in air or soil^8^. Thus, this closer contact with the surrounding water likely affects early gut colonization. Whilst water seems to affect the fish gut microbiota already from mouth opening onwards^9^, feed microbiota becomes important at a later development stage^10^,^11^. However, although water and feed are the two main sources of microorganisms available to fish, the factors underlying the successful colonization of ingested microbes and the community assembly inside the gut are still poorly understood^12^. For this reason, predictability and repeatability of gut microbiota manipulations is currently limited.

The nutrient rich environment of high density aquaculture is conducive to the proliferation of microbes. To this end, aquaculture management aims to minimize the amount of nutrients in the rearing tank. For example, in recirculating aquaculture systems (RAS) the first step of water purification is solids removal^13^,^14^. In contrast, in active suspension (AS) systems, fish excreta and feed left overs are mineralized inside the rearing tank. AS systems are characterized by the formation of bioflocs (aggregates of microorganisms, colloids, organic polymers and dead cells), which contribute to the maintenance of good water quality while providing additional nutrients to the fish^15^.

In the present study, we hypothesized that water bacterial communities would differ between the two rearing environments (i.e. RAS vs. AS systems) due to differences in systems’ design. Assuming that gut colonization is strongly affected by the composition of the microbiota in the water, we expected that composition of gut communities would differ between systems as well. Compared to RAS, bacteria in the water of AS systems have a higher chance of being successfully transferred in the gut, due to the constant grazing of larvae on bioflocs. Thus, a greater influence of AS water microbiota on the gut is expected. We used 454 pyrosequencing of PCR-amplified bacterial 16S ribosomal RNA (rRNA) gene fragments to characterize for the first time the composition of gut BCs of Nile tilapia *Oreochromis niloticus* (Linnaeus) reared for 42 days in RAS or AS tanks from the moment of first feeding. The progressive gut colonization in fish reared in the two systems and the similarity of water and feed BCs with those in the gut were evaluated at different time points. Furthermore, the potential role of the most predominant bacteria found in this study is discussed in detail based on a comprehensive literature review.

## Materials and Methods

### Experimental animals and set up

The experiment was performed in accordance with relevant guidelines and approved by the Ethical Committee of Wageningen University for animal experiments (Registration code: 2009055d). One batch of three days post-fertilization (3 dpf) eggs were washed out from the mouth of a female Nile tilapia and incubated for four days at 27 °C. Eggs were incubated in a tank until swim-up stage and received water from the same source. For rearing the larvae, two different culture systems were used: a recirculating aquaculture system (RAS) and an active suspension (AS) system with two replicates each (Ra&Rb and AS1&AS2), which were initially filled with the same water coming from a well. Replicate systems did not share the same water. Only for RAS systems, each replicate also contained 2 replicate tanks (Ra1, Ra2 & Rb1, Rb2) which were connected to the same water treatment unit (biofilter). In each tank, 100 randomly selected swim-up larvae (7 dpf) were introduced before first feeding and before mouth opening. Mouth opening occurred within the experimental rearing systems. Feeding started 9 dpf (referred to as day 0) and was continued for 42 days. Fish were fed three times a day to apparent satiation with a starter feed (F-0.5 GR Pro Aqua Brut – Trouw Nutrition, France; 57% crude protein, 15% crude fat, 8.5% carbohydrates, 11% crude ash, 0.6% crude fiber, 1.7% phosphorus). A 42 day culture period was considered sufficiently long for all major stages of the gut development^16^. Water, feed and gut samples were collected on day 7 and 42. Further details about the experimental setup, animals and description of the rearing systems can be found elsewhere^17^.

### Sampling of guts, water and feed

On sampling days, three tilapia larvae were randomly collected from Ra1, Ra2, Rb1, Rb2, AS1 and AS2 for gut BC analysis. After euthanization, the larvae were rinsed with 70% ethanol and sterile water, before dissecting the gut. Gut samples were flash-frozen in liquid nitrogen and stored individually at −80 °C until further processing. Furthermore, 250 mL of water was collected from each tank. Water was simultaneously filtered through a 0.45 μm (type HAWP) over a 0.22 μm (type GTTP) membrane filter (Millipore - Isopore™) using a vacuum apparatus. Filters were stored at −80 °C until processing. The 0.45 μm filters were used in order to avoid clogging of the 0.22 μm with large size particles during filtration. However, both filters were used for DNA extraction from water. Finally, also 2 grams of feed were collected and stored at −80 °C until processing. All gut, water and feed samples were stored and analysed individually. Samples were not pooled neither were the corresponding DNA extracts.

On a weekly basis, total ammonia nitrogen (TAN-N), nitrite (NO_2_^−^-N), nitrate (NO_3_^−^-N), orthophosphate (PO_4_^3−^), carbon dioxide (CO_2_), urea, dissolved oxygen (DO), pH, conductivity and temperature (°C) were measured in each tank according to Giatsis *et al*. (2014).

### Isolation of DNA

DNA was extracted from larval gut samples using the DNeasy Blood & Tissue Kit (Qiagen, Venlo, Netherlands), whereas the FastDNA SPIN kit for soil (MP Biomedicals, Ohio, USA) was used for water and feed samples. The use of different physical, mechanical, chemical and enzymatic lysis protocols, different purification and precipitation techniques and different reagents, have shown to affect genomic DNA yield and quality. This was confirmed by Lever *et al*.^18^, however, these authors furthermore concluded that the observed effects are almost always sample dependent. This being said, before our study, genomic DNA was extracted from all sample types by using two extraction protocols. Since the DNeasy Blood & Tissue Kit resulted in a very low DNA yield from the water and feed samples and a high DNA yield was obtained with the FastDNA SPIN kit for soil, and hence the latter was used for all water and feed samples in this study. In contrast, the FastDNA SPIN kit for soil showed a low yield for DNA extraction from gut samples, while a high yield was obtained with the DNeasy Blood & Tissue Kit. Therefore, the DNeasy Blood & Tissue Kit was used for all gut samples in this study.

Modifications of the manufacturer’s protocol with respect to enhancement of cell lysis (i.e. bead beating), DNA purification and elution processes, were made as previously described by Giatsis *et al*. (2014).

### PCR and 454 Pyrosequencing

Barcoded amplicons from the V1-V2 region of 16S rRNA genes were generated by PCR using the 27F-DegS primer^19^ appended with titanium sequencing adaptor A and an 8 nt sample-specific barcode^20^ at the 5′ end. As reverse primer, an equimolar mix of two primers 338R I and II^21^,^22^ was used to carry the titanium adaptor B at the 5′ end. A detailed protocol for the PCR is given by Giatsis *et al*. (2014). Nucleotide sequences were generated by pyrosequencing using an FLX genome sequencer in combination with titanium chemistry (GATC-Biotech, Konstanz, Germany). These sequence data were submitted to the European Bioinformatics Institute under study accession No PRJEB4462 and sample accession No ERS343984 – ERS344037.

### Data handling

Pyrosequencing data were analyzed using the QIIME 1.5.0 pipeline^23^ and quality filtering was performed as follows: low quality sequences were removed using default parameters. Removed reads included (i) reads with less than 200 or more than 1000 nucleotides; (ii) reads with more than 6 ambiguous nucleotides, homopolymer runs exceeding 6 bases, reads with missing quality scores and reads with a mean quality score lower than 25; and (iii) reads with mismatches in the primer sequence. Operational taxonomic units (OTUs) were identified at the 97% identity level. Representative sequences from the OTUs were aligned using PyNAST^24^. The taxonomic affiliation of each OTU was determined using the RDP classifier at a confidence threshold of 80% against the 12_10 Greengenes core set^25^. Possible chimeric OTUs were identified using QIIME’s ChimeraSlayer and removed from the initially generated OTU list, producing a final set of non-chimeric OTUs.

To visualize possible commonalities and anti-correlations between gut, water and feed BCs, physical samples and OTUs were plotted as nodes in a bipartite network. In the network analysis, the average relative abundance of OTUs in replicate samples was represented. To cluster the OTUs and hosts in this network, the Edge-weighted Spring Embedded algorithm as implemented in Cytoscape 3.0.2 was used^26^.

### Statistical analysis

A repeated measure ANOVA was applied for water quality measurements on day 7 and 42 with “system” (RAS or AS) as main factor and “day” (7 or 42) as repeated measure. Based on Draftsman plots (variable pair-wise scatter plots) of water quality data, the TAN values were log-transformed (Figure S1). Subsequently, all environmental variables were normalized. The Euclidean distance of normalized environmental variables was used as dissimilarity measure. Gut, water and feed BC data were expressed as relative abundance of all OTUs in each sample, and Bray Curtis similarity was calculated based on square root transformed data.

A distance-based linear model (distLM) was used for analysing and modelling the relationship between the bacterial community composition, as described by a resemblance matrix, and the predictor variables (i.e. water parameters). When not enough possible permutations were available, P-values for testing the null hypothesis of no relationship between the two datasets were obtained by Monte Carlo permutations (a sample was drawn from the theoretical asymptotic permutation distribution)^27^. Forward selection of the environmental variables was applied and distance-based redundancy analysis (dbRDA) was used for visualization^28^,^29^.

Based on Bray Curtis similarity of relative abundance data, RELATE analysis evaluated the relatedness between water and gut BC by calculating Spearman’s ρ correlation coefficient between all elements. Similarity percentages (SIMPER) analysis was used to determine the OTUs which contributed most to the discrimination or relatedness of samples in each group. To measure alpha-diversity of the bacterial communities: the total number of observed species (S), Pielou’s evenness (J′ = H′/log(S)) and Shannon (H′ = −SUM(P_i_*ln(P_i_))) were calculated (P_i_ is the proportion of the total count (abundance) arising from the *i*th species). As species richness and evenness can only be compared between samples when sample sizes are equal^30^, resulting reads were randomly selected so as to standardize to the sample with the least number of obtained reads (n = 1719). To visualize community evenness, dominance plots were created based on relative abundance data. OTU relative abundance (y-axis) was averaged per sample type (gut and water), for each system (AS and RAS) per day (7 and 42). Cumulative relative abundance was plotted against the increasing species rank (x-axis).

Statistical analyses were performed using Primer 6 (version 6.1.11) (Primer-E Ltd., Plymouth, United Kingdom) and its PERMANOVA add-on package^31^. Venn diagrams were produced in *Venny* online freeware^32^.

### Phylogenetic analysis

The first 150 OTUs contributing most to differences between groups were considered for a more thorough phylogenetic analysis. Representative sequences of these OTUs were aligned using the online SINA alignment service of the ARB-Silva database^33^. Aligned sequences were imported into ARB in the Silva release 115 SSU NR 99 tree^34^ using the ARB parsimony method without changing the tree topology. Three near neighbors of all 150 OTUs were selected according to the following criteria: (i) they were at least 800 nucleotides long and included the entire sequenced amplicon, and (ii) neighbors per OTU were picked from different published studies. An alignment containing only the neighbors was exported before constructing a Bayesian tree. Ambiguous regions of the alignment were systematically removed using the program Gblocks v.0.91b^35^. Default parameters were used, except allowing a minimum block length of 5 and gaps in 50% of positions. Phylogenetic trees were calculated by Bayesian analysis, using a locally installed version of MrBayes 3.2^36^. All parameters were treated as unknown variables with uniform prior-probability densities at the beginning of each run, and their values were estimated from the data during the analysis. All Bayesian analyses were initiated with random starting trees and were run for 10^7^ generations. The number of chains was set to four and Markov chains were sampled every 1000 iterations. Points prior to convergence were determined graphically and discarded. Calculated trees were imported into ARB, and short sequences obtained in this study were subsequently added by use of the ARB parsimony method without changing the tree topology.

## Results

### The effect of rearing system and time on water quality

Analysis of environmental data showed significant differences in water quality between RAS and AS. Conductivity, NO_3_-N and PO_4_-P were higher, whereas CO_2_, DO and pH were lower in RAS (P < 0.05) than in AS (Table 1). Conductivity, NO_3_-N and PO_4_-P increased, and CO_2_, DO and pH dropped between day 7 and 42 (P < 0.05). In contrast, temperature and concentrations of NO_2_-N, urea and TAN were neither significantly different (P > 0.05) between RAS and AS, nor between day 7 and 42.

### Correlation of environmental parameters with water bacterial communities

**Figure 1:**
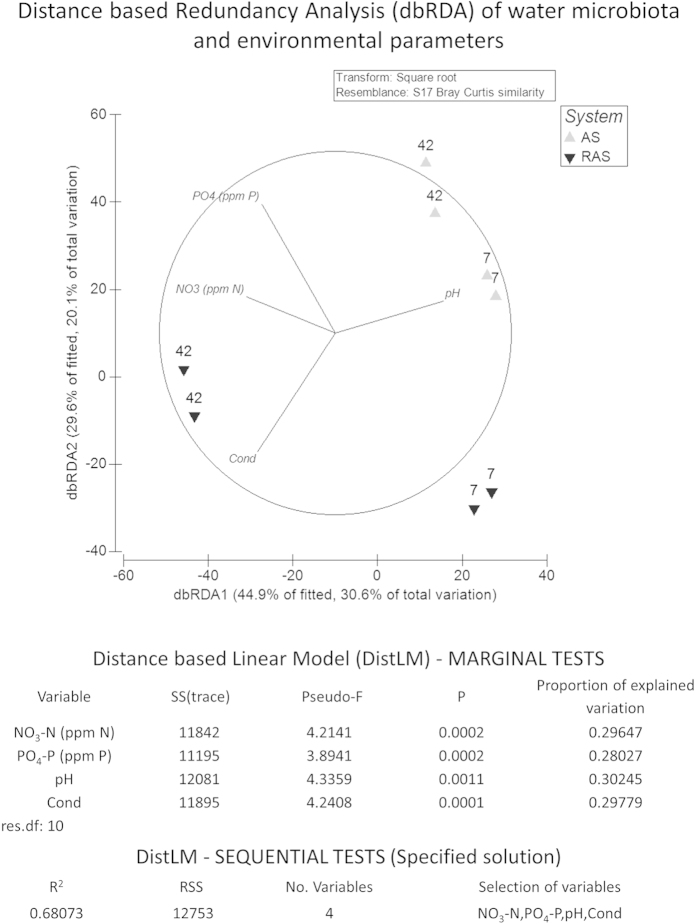
Distance based Redundancy Analysis (dbRDA) of water microbiota. Relative position of water samples in the biplot is based on Bray Curtis similarity of square root transformed relative abundance at the OTU level. Vectors indicate the weight and direction of those water quality parameters that were best predictors of water bacterial composition as suggested by the results of the distance-based linear model (distLM). The dbRDA axes describe the percentage of the fitted or total variation explained by each axis while being constrained to account for group differences. Sample IDs indicate the sampling day (7 and 42) and the rearing system (AS & RAS: Active suspension and recirculating aquaculture system with two replicates each). NO_3_^−^-N: Nitrate nitrogen; PO_4_^3−^-P: Phosphate phosphorous; Cond.: Conductivity.

Distance-based linear model (DistLM) analysis revealed that pH, conductivity, NO_3_-N and PO_4_^3^-P together explained 68% (R^2^ sequential) of the observed total variation in the composition of the bacterial community in the water. Conductivity and pH strongly correlated with differences in BCs between RAS and AS, while PO_4_-P and NO_3_-N correlated with differences in the communities between day 7 and 42 within system (Fig. 1). The same parameters showed a highly significant system * day interaction with repeated measures ANOVA of water quality data (Table 1).

### Similarity of water bacterial communities between replicate systems over time

Overall, 11,658 OTUs were identified in all samples. At OTU level, the average similarity of water BC within AS on day 7 was 64% (Table 2). The 5 OTUs (SIMPER analysis), in order of relative abundance, explaining most of the average similarity were OTU 9828 (order Sphingomonadales), 4552 (genus *Limnohabitans*), 8066 (genus *Sediminibacterium*), 2756 (genus *Rhodobacter*) and 11945 (genus *Nitrospira*). Combined, these 5 OTUs accounted for 49% of the bacterial community in AS water on day 7 (Table S1).

On day 42, the water BC average similarity within AS decreased to 26% (Table 2). Almost 60% of this similarity was attributed to OTU 8066 (genus *Sediminibacterium*), 14254 (family Comamonadaceae), and 4686 (order Sphingobacteriales) (Table S1). Other predominant OTUs of AS water BC on day 42 included: OTU 5688 (genus *Fimbriimonas*) and 7261 (genus *Armatimonas*).

Overall, water bacterial communities in AS systems on day 7 was 17% similar with BC present on day 42 since most of the predominant OTUs on one day were absent or rare on the other (Table 2). Despite the low similarity, 85 OTUs were shared between the two dates, with a cumulative relative abundance of 63% on day 7 and 52% on day 42 (Table 3). OTU 8066 contributed most to the similarity, whereas OTU 9828 contributed most to the dissimilarity between the two days (Table S1).

In RAS, the average similarity of water BC on day 7 was 59% (Table 2). More than half of this similarity was attributed to only two predominant OTUs: OTU 7333 (genus *Polynucleobacter*) and 9828 (family Sphingomonadales), which together had a cumulative relative abundance of 46.8% on that day (Table S1).

On day 42, similarity of water BC within RAS remained at levels similar to day 7. This similarity was mostly attributed to OTU 7090 (family Rhodocyclaceae), 14286 (genus *Limnohabitans*), and 4377 (genus *Rhodobacter*). Other predominant OTUs of RAS water BC on day 42 included: OTU 5688 (genus *Fimbriimonas*) and 7261 (genus *Armatimonas*). The aforementioned five OTUs accounted for 55.2% of the total bacterial community on that day (Table S1).

Bacterial communities in the RAS water differed considerably between day 7 and 42 (3.5% similarity) (Table 2). Predominant OTUs in water on day 7 were almost absent on day 42 and vice versa. For example, OTU 7090 was absent on day 7 whilst accounted for 35% of the community on day 42, thus contributing about 20% to the difference between the two days (Table S1). Despite the low similarity, water BC in RAS shared 98 OTUs between day 7 and 42, with a cumulative relative abundance of 76% and 57%, respectively (Table 3).

### Similarity of water bacterial communities between systems

Already on day 7, the average similarity between AS and RAS was only 24% (Table 2). The two systems shared 113 OTUs with a cumulative relative abundance of 62.5% and 73% in AS and RAS systems, respectively (Table 4). On day 42, the similarity of water BC between the two systems decreased to 2%, since almost all predominant OTUs in AS water, were absent in RAS and vice versa. Overall, water from the two systems shared 40 OTUs with a cumulative relative abundance of 30% in AS and only 4% in RAS (Table 4).

In terms of evenness, an overall system (AS & RAS) * day (7 & 42) interaction was observed. On day 7, 17 OTUs had a cumulative relative abundance of 60% in AS water, whereas on day 42, the same percentage was attributed to six OTUs. In RAS water, 60% of the relative abundance was attributed to 6 OTUs on day 7 and three OTUs on day 42 (Table S1). Additionally, over time species richness decreased from 683 to 394 OTUs in AS water and from 815 to 552 in RAS. This observation is also in agreement with the results of the dominance plots and the biodiversity indices which showed that the richness and evenness of water bacterial communities in AS decreased between day 7 and 42, whereas this difference was less pronounced in RAS (Figure S2 & Table S2).

### Similarity of gut bacterial communities between replicate systems over time

On day 7, average similarity of gut bacterial communities within AS was 69% (Table 2). Half of this similarity was attributed to OTUs 4790 (species *Mycobacterium llatzerense*), 1651 (genus *Rhodobacter*), 10562 (genus *Gordonia*), 10599 (family Bradyrhizobiaceae) and 8299 (genus *Sporocytophaga*). Those five OTUs accounted for a cumulative relative abundance of 42.2% in AS (Table S1). On day 42, the similarity of gut BC between samples dropped to 60%. More than 80% of this similarity was attributed to OTUs 4563 (family Isosphaeraceae), OTU 12537 (genus *Arthrobacter*) and OTU 4790 (species *Mycobacterium llatzerense*). Those predominant OTUs were encountered on both days, albeit at different relative abundances (Table 5). *M. llatzerense* (OTU 4790) was the only OTU with a high relative abundance on both sampling days. Despite the 131 shared OTUs between the two days, gut BC was 21.6% similar between days (Table 2, Table 3).

Within RAS, average similarity of gut BC between samples was 55% on day 7 (Table 2). More than 60% of this similarity was due to OTUs 10599 (family Bradyrhizobiaceae), 1005 (genus *Rhodococcus*), 4790 (*Mycobacterium llatzerense*) and 12555 (genus *Agrococcus*) (Table S1). On day 42, average similarity between gut samples remained similar. This similarity was mostly attributed to OTUs 7966 (family Peptostreptococcaceae), 4790 (*Mycobacterium llatzerense*), 7955 (family Mogibacteriaceae), 1005 (genus *Rhodococcus*) and 2787 (*Cetobacterium somerae*). These five OTUs accounted for a cumulative relative abundance of 59.5% in RAS. All predominant gut OTUs of day 7 were also present on day 42, albeit at lower relative abundance, whereas the OTUs most predominant on day 42 were absent on day 7 (Table S1). Similar as for AS, *M. llatzerense* was the shared OTU with a high relative abundance on both sampling days. In RAS, 302 gut OTUs with a cumulative relative abundance of 91% and 51% respectively, were shared between days (Table 3).

### Similarity of gut bacterial communities between systems

On day 7, similarity of gut BC between systems was 22% (Table 2). Most of the predominant OTUs were present in both systems, although at different relative abundance (Table 5). OTUs 1651 and 8299 were predominant in AS but absent in RAS and OTU 3934 was predominant in RAS though absent in AS. Despite the low similarity in gut BC, the two systems shared 247 OTUs with a cumulative relative abundance of about 71% in both systems (Table 4).

On day 42, similarity between systems decreased to 11% (Table 2). This was partly due to the difference in the abundance of OTU 4563 (family Isosphaeraceae) between the two systems (40.8% in AS and absent in RAS) (Table 5). Among the shared OTUs on that day, *M. llatzerense* was the OTU with high relative abundance in both systems. Overall, the number of shared OTUs between systems decreased over time from 247 to 90 (Table 4).

In terms of biodiversity of the BCs, a system (AS & RAS) * day (7 & 42) interaction was observed. On day 7, 60% of cumulative relative abundance in AS was attributed to 13 OTUs whereas on day 42 to only two; this is clearly depicted also in the large decrease of the Shannon index over time (Table S2). On the contrary, in RAS, the 60% of the abundance was attributed to five and six OTUs on day 7 and 42, respectively (Table S1) while the biodiversity indices remained on similar levels. Over time, in AS, the total number of observed gut species decreased from 732 to 279, whereas this decrease was not so evident in RAS. This observation is in agreement with the results of the dominance plots for day 7 (Figure S2).

### Predominant OTUs of feed bacterial communities

Since only one feed sample was analyzed per sampling day, no statistical comparison between sampling days was performed. OTUs 5872 (genus *Corynebacterium*), 14212 (genus *Photobacterium*), 3747 (genus *Sporosarcina*), 2650 (genus *Psychrilyobacter*), 3532 (genus *Facklamia*) and 1521 (species *Anoxybacillus kestanbolensis*), were among the most abundant OTUs on both days (Table S1, Table 5).

### Relationship between gut, water and feed bacterial communities

**Figure 2:**
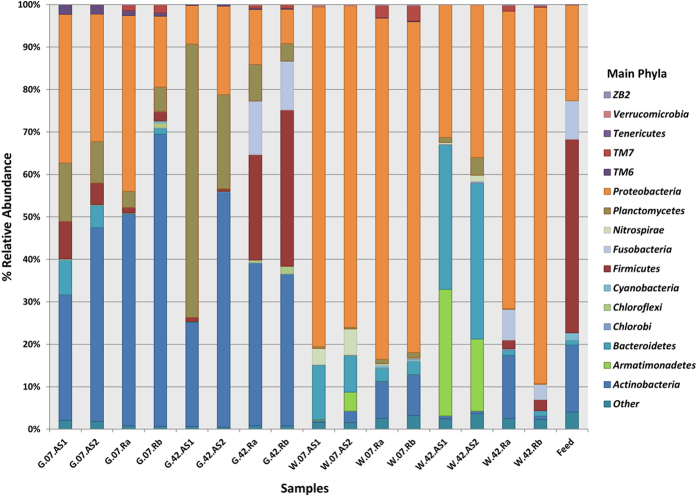
Cumulative bar charts of the main phyla present in either gut or water samples. Percentages show the relative abundance of each phylum in the gut or water of each replicate system on either day 7 or 42. Phyla in the feed represent the average values of feed samples from both day 7 and 42. AS1 & AS2: Replicate active suspension system 1 and 2, Ra & Rb: Recirculating aquaculture system a and b.

RELATE analysis revealed a strong correlation (Spearman rank correlation Rho 0.807, P < 0.0001) between bacterial communities in gut and water (Table S3). In other words, differences among water and gut samples followed similar patterns, even though the bacterial community composition in gut and water was different (P < 0.001). On day 7, Proteobacteria dominated the water of both systems. In RAS, Proteobacteria was also the dominant phylum on day 42, but Fusobacteria and Firmicutes, which were absent on day 7, appeared. In AS systems, Bacteriodetes and Armatimonadetes replaced a large fraction of the Proteobacteria by day 42. Feed-associated BC was dominated by Firmicutes and Proteobacteria, followed by Actinobacteria and Fusobacteria (Fig. 2).

**Figure 3:**
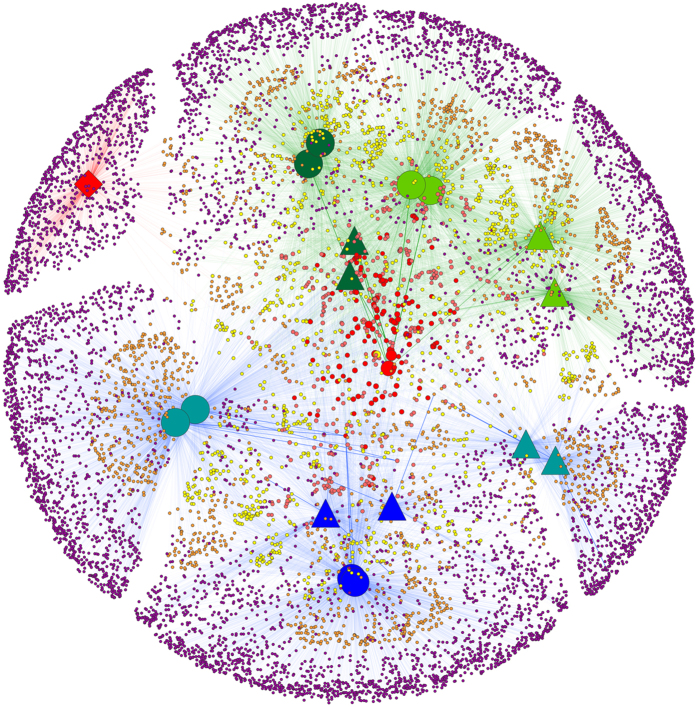
Network-based analyses of gut, water and feed bacterial communities. The network diagram is color-coded by: sample type (gut: green, water: blue and feed: red); day 07 (circles), day 42 (triangles) and feed which represents a pooled sample both from day 7 and 42 (square); system (gut AS: Active suspension system with two replicates: dark green; gut RAS: Recirculating aquaculture system with two replicates: light green; water AS: Active suspension system with two replicates: dark blue; water RAS: Recirculating aquaculture system with two replicates: light blue). Sample nodes are oversized to be distinguished from OTU nodes. Colour and size of OTU nodes represent assignments of OTUs to sample nodes based on the amount of samples that were shared with (small nodes are shared with less samples compared to larger ones); purple OTU nodes: OTUs present in only one sample; orange OTU nodes: OTUs shared between two samples; yellow OTU nodes: shared between 3 or 4 samples; pink OTU nodes: shared between 5 to 7 samples; red OTU nodes: shared between 8 to 16 samples). Thickness/brightness of edges connecting two nodes is proportional to the relative abundance of the specific OTU in the physical sample to which it connects.

In spite of a high inter-sample correlation, average similarity between gut and water bacterial communities in AS was between 4% and 8% for both days (Table 2). This was mostly due to the low number of subdominant OTUs shared between gut and water (Fig. 3). This low similarity between gut and water on day 7 was attributed to 41 shared OTUs which represented 50% of the cumulative relative abundance in gut and 29% in water. On day 42, 40 shared OTUs between gut and water had a cumulative relative abundance of 90% and 40%, respectively (Table 6).

In RAS, similarity between gut and water BC was 4 and 8% in the two day respectively (Table 2). Similarity on day 7 was attributed to 155 shared OTUs with a cumulative relative abundance of 83.5% and 20%, in gut and water respectively. On day 42, the 86 shared OTUs had a cumulative relative abundance of 81% and 12% (Table 6).

On the two days, gut shared with feed ten and five OTUs in AS and eight and three in RAS (Table 6). For both systems and days, the similarity between gut and feed was below 1% (Table 2). In AS, OTU 5872 was the only dominant OTU in feed encountered in the gut on day 7. Overall, only two OTUs were shared among gut, water and feed bacterial communities in AS or RAS on day 7, and only one OTU on day 42 (Table 6).

## Discussion

The higher levels of nitrate observed in RAS compared to AS could be due to the higher nitrification rates. In RAS, through the water re-use loop, settable solids consisting mainly of organic matter, are removed before aerobic chemoautotrophic nitrifying bacteria convert ammonia via nitrite into nitrate in the biofilter^13^,^14^,^37^. In AS, solids were not removed from the fish rearing unit, but maintained in suspension. The accumulating organic matter in AS was used as substrate for biofloc formation^15^,^38^,^39^,^40^. Heterotrophic bacteria in bioflocs immobilize NH_3_-N, reducing the need for nitrification. Nitrification concurs with reduction of alkalinity. This explains why in AS the pH was higher than in RAS. However, the lower pH in RAS did not result in a higher CO_2_ level, because the amount of free CO_2_ in a system depends on the total amount of carbon dioxide/bicarbonate/carbonate (i.e. the total alkalinity) present^41^.

In AS systems, a nitrification-denitrification coupling might have contributed to the observed lower NO_3_-N concentrations^42^,^43^. Nitrification occurs in the aerobic surface layer of bioflocs, whereas denitrification can take place in deeper anoxic layers^44^,^45^,^46^. The high relative abundance of *Nitrospira* in water bacterial communities of AS on both days, suggests the occurrence of nitrification in those systems. *Nitrospira* is an aerobic, lithoautotrophic, nitrite-oxidizing bacterium that is commonly encountered in aquaculture systems or in freshwater activated sludge particles^47^. However, this genus exhibits high substrate affinity and has been mainly reported in filter materials, bio-filters of freshwater, brackish and marine systems^37^,^48^,^49^,^50^,^51^.

The second most abundant OTU (14254) in AS water of day 42 belonged to the Comamonadaceae family. Members of this family play a role in nitrate removal as well as in poly-hydroxy-alkanoate (PHA) metabolism. This indicates the metabolic and regulatory relationships between PHA degradation and denitrification by PHA-degrading denitrifiers in anoxic environments^52^,^53^,^54^,^55^.

The PO_4_-P concentration in RAS has been higher than in AS. Microbial phosphate assimilation was suggested as the main cause of the lower phosphate levels in biofloc systems when compared to those in RAS^42^,^56^,^57^. The most predominant OTU in RAS water on day 42 was a member of family Rhodocyclaceae. This family contains mainly aerobic or denitrifying bacteria which live in aquatic habitats and exhibit very versatile metabolic capabilities. Rhodocyclaceae species are commonly reported in waste water where they play an important role in bioremediation (e.g. phosphate removal)^58^,^59^,^60^,^61^,^62^,^63^. Information at genus level on its role in phosphorus cycling would be needed to explain differences in abundance between AS and RAS.

DistLM analysis revealed a high correlation between water quality parameters and the bacterial communities present in the water. A high fraction of the total variation (68%) in water BC was explained by pH, NO_3_-N, PO_4_-P and conductivity. Ebeling *et al*. 2006, described under which conditions autotrophic, heterotrophic, nitrifying or denitrifying processes dominate in aquatic systems^41^. In fact, differences in water quality between RAS and AS were caused by differences in system design and management, which induced differences in composition and functionality of water BC and vice versa. Although the percentage of explained variation in water BC by environmental factors was high, it remains challenging to control bacterial communities composition, species dynamics and functionality because: (a) possibly other factors, presently not measured, are better “predictors” of the bacterial communities and (b) there is a high degree of stochasticity involved in how bacterial communities structures change over time, even in replicated systems^17^. In addition, interpretation of the DISTLM results should be done with care, especially when sample size is low. Despite the fact that permutation methods allowed us to reach the desirable significance level to test our hypothesis (i.e. correlation between water quality and water microbial communities), the high percentage of explained variation does not directly imply causality between the two datasets, but correlation. Future research, including more systems and data points should be performed in order to verify if the observed correlations are maintained.

The time * system interaction for water BC revealed very interesting trends. First, the similarity of water BC between replicate AS tanks decreased over time, whereas in RAS it remained stable. This suggests that sustainable “replication of water bacterial communities” is more feasible in RAS than in AS. Secondly, the average similarity of water BC between day 7 and 42 in AS was higher than in RAS. Although in RAS more OTUs were shared between days, water bacterial communities in AS remained five times more similar over time compared to that in RAS. This observation could be partly explained by some predominant OTUs in the water, which over time, were more robust in AS than in RAS. For example, *Sediminibacterium* (OTU 8066) in AS water was among the most predominant OTUs on both days. This genus contains two species exhibiting high versatility in types of used substrates including non-starch polysaccharides (NSPs), oligosaccharides and others. These species were found in lakes, freshwater reservoirs, activated sludge reactors, stream biofilms and nutrient rich environments^64^,^65^,^66^,^67^,^68^,^69^,^70^.

Water BCs similarity between AS and RAS systems decreased considerably over time, as shown by the noticeable (from 247 to 90) decrease of the number of shared OTUs (Table 4). On day 7, the predominant water OTUs of either AS or RAS were found in both systems though in different relative abundances. On day 42, the predominant OTUs in water of AS systems were not encountered in RAS, and vice versa. Both systems were initially inoculated with water from the same source, and thus with a similar BC, which shows that the system design and function induced the development of a system-specific specialized bacterial community.

It is important at this point to remember that regarding water BCs in particular, the observations and patterns discovered between the two systems, or over time, are just a useful first starting point. This is firstly because in some cases sample size was low (especially for within system comparisons), though high enough to allow statistical power. Secondly, the environments in this study, although relevant to aquaculture are not natural since they operate under controlled conditions. This means that it is uncertain whether the observed patterns would occur in natural habitats or for example in an extensive outdoor aquaculture system (e.g. a fish pond). Nevertheless, this work can be the basis of future work addressing the implications that rearing systems have on water and fish gut BCs and provide awareness on system microbial management in aquaculture.

The described differences in composition of water BC between the two systems give rise to the question whether these differences had an impact on gut communities, and of course to which extent. The assumption has been that water provides the first microbial colonizers from the moment of mouth opening until first feeding, whereas feed microbiota would dominate during later development^9^,^10^,^11^. Bakke *et al*. 2013, suggested that relatively small differences in water BC might impose significant differences in gut BC of fish larvae^71^. In line with this suggestion, our results showed a high and consistent correlation between the bacterial community composition in water and gut (Spearman’s ρ: 0.807).

Although the similarity of water BC between replicate AS decreased over time, the average similarity of gut BC decreased little. Similarly in RAS, a significant decrease in average similarity of water BC over time was not observed in gut which remained eight times higher in gut than in water (Table 2). A considerable number of gut OTUs was shared between day 7 and 42. For example in RAS, OTU 1005 (genus *Rhodococcus*) was a predominant OTU in the gut on both days. The genus *Rhodococcus* has been reported in gut microbiota of sole, red rock fish, Norwegian mackerel, USA smelt, rainbow trout and shrimp^72^,^73^,^74^,^75^.

The relatively larger effect of time on water communities suggests that water BC were more sensitive to environmental changes than bacterial communities residing in the gut of fish larvae. To a certain degree, microbial community stability highly depends upon potential disturbances of the two ecosystems i.e. the gut and the water, as well as the ability of a community to respond resiliently. Preservation of homeostasis at the intestine should be in the gut microbiota’s best interest, in order to provide a convenient long-term habitat. In favor of both, host also contributes to a fairly stable environment leading to a homeostatic interaction between host and gut microbiota. However, during early fish development, rapid anatomical, physiological and immunological changes occur in the gut, which makes it plausible to think that equally substantial changes should have also occurred in the gut communities. Fernandez *et al*.^76^ evaluated the behavior of different microbial communities under perturbed conditions and suggested that stability of the functionality does not necessarily imply stability of community structures. This means that in our study, observed changes in the gut community structure over time, could have occurred side-by-side with changes in community functionality. In the same line of thought, observed changes of water physicochemical properties might have been responsible for the relative change of water BCs over time, but whether the latter compromised functional stability of the community, is not known. Further research is required to elucidate the relationship between microbial community structure (i.e. composition, diversity) and its ability to organize in a way that assures the ability of counteracting the stress effects, by maintaining functionality.

Regarding predominant species, an interesting system * day interaction was observed in the gut communities. On day 7, the number of observed gut species in the AS systems was higher than in RAS. This higher richness coincided with higher gut community evenness i.e. higher biodiversity. On day 42 a considerable decrease both in the community richness and evenness was observed in AS. The latter means that a smaller fraction of the different species was present in dominant numbers whereas this was not so evident for fish reared in the RAS systems. Based on our data, it is not possible to clarify with certainty the reason for the observed differences between the two systems. Nevertheless, the fact that similar temporal changes in community biodiversity were also observed in water BCs might point out the role of water as a microbial reservoir available to the fish gut. However, more studies would be necessary to test how changes in community structure of a habitat impact gut microbial biodiversity of the organisms living therein and whether such changes affect the functionality of the gut community.

Similarity of gut bacterial communities between AS and RAS was overall low and further decreased over time. On day 7, OTU 1651 (genus *Rhodobacter*) was a predominant OTUs in the gut of AS, but absent in RAS. The genus *Rhodobacter* contains Gram-negative bacteria widely distributed in fresh water as well as marine and hypersaline habitats (Table S4, Figure S3). *Rhodobacter* species have been reported in the gut of grass carp, rainbow trout, Siberian sturgeon, catfish, Atlantic cod and Pacific white shrimp^77^,^78^,^79^,^80^,^81^,^82^,^83^, as well as in the water of aquaculture systems^84^,^85^.

A similar pattern was observed for the gut BC in RAS. OTU 7966, (family Peptostreptococcaceae) was predominant in the gut on day 42, absent on day 7, and present at very low relative abundance levels in AS. This suggests that both systems and developmental stage of the fish influence the presence and abundance of this OTU. Peptostreptococcaceae is a family of obligate anaerobic bacteria^86^. In salmon, Peptostreptococcaceae was detected as a member of both the allochthonous and autochthonous microbiota regardless of the fish diet, indicating that Peptostreptococcaceae might be a natural part of the intestinal microbiota of this species^87^. The family has been also reported in the gut microbiota of yellow catfish^88^,^89^ and common carp^90^.

In this study, out of 11,658 OTUs, two OTUs were detected in all gut and water samples. Of these, *Mycobacterium llatzerense*, a member of the phylum Actinobacteria, was present at high relative abundance in all gut samples, and less abundant in water samples. The second OTU, a member of the Bradyrhizobiaceae, was among the predominant gut OTUs on day 7 for both systems, less abundant on day 42, and also present in all water samples. Whether its presence in the water was due to fish defecation or whether it originated from water and managed to proliferate in the fish gut is presently not known.

*M. llatzerense*, has not been previously reported in aquaculture systems but has been reported in hemodialysis water, and drinking water production and distribution systems^91^. The association of *M. llatzerense* with groundwater (Table S4, Figure S3) may reflect a more psychrophilic profile of this species compared to other Mycobacteria, and might justify its presence in our systems which rely on well water. Although *M. llatzerense* has not been previously reported in fish gut microbiota, the high and consistent abundance in all guts suggests the preference of these bacteria for the gut environment of tilapia larvae. *M. llatzerense* is facultative autotrophic, aerobic, non-fermentative, and involved in hydrogen oxidation and alkaline dephosphorylation^92^.

Members of the Bradyrhizobiaceae family have been found in sewage treatment plants^93^, municipal wastewater treatment plants^94^, active granular activated carbon filters and drinking water distribution systems (Table S4, Figure S3)^95^. This is in agreement with the presence of Bradyrhizobiaceae in all water samples of this study. In fish, the Bradyrhizobiaceae family was overrepresented in the gut microbiota of sea bass^96^, and was also reported in the stomach of yellow catfish^88^. Since the family contains more than ten different genera, it is difficult to speculate about the role and functionality of these microorganisms in the fish gut.

More information and relevant literature regarding major functions and processes as well as environment preference of the encountered OTUs are provided in Table S4 of the supplementary material.

Interestingly, the OTUs shared between gut and water had a higher relative abundance in the gut than in the water. In turn, almost none of the dominant OTUs in water were detected in the gut. This could suggest that host-specificity for particular microbial species is modulated by selective pressures within the host gut attributed to gut habitat (i.e. physiology, anatomy) and host’s genotype^97^,^98^,^99^. On the other hand in water, low abundance of the predominant gut OTUs can be partly explained by fish defecation. Apparently, conditions in water are suboptimal for the growth of defecated bacteria mostly due to the ecological preference of the latter for the gut habitat (i.e. pH, anoxic conditions, etc.), adhesion sites and nutrients availability therein. Despite the significant effort in defining the forces which impact the assembly of gut microbial communities, the underlying mechanisms attributed to the impact of the environment over the host selectivity are still poorly understood.

Feed predominant bacteria were mostly absent in water and gut. Only 1% of gut OTUs was shared with feed at very low relative abundances. It is important at this point to mention that these results do not underestimate the importance of feed per se on gut microbial communities. Feed composition, nutrients, production technology, etc. have been all found to affect gut microbiota in mammals^7^,^100^,^101^,^102^,^103^,^104^ and fish^87^,^105^,^106^,^107^. This is not the case in our study where the same feed was used in all treatments. We put forward that the low number of subdominant bacteria shared between the feed and the gut indicates the low influence that feed communities had in the gut compared with those present in the water. Bakke *et al*. 2013, working with cod larvae, suggested that microbiota in water had a stronger influence than microbiota in the feed on the microbial community composition in the gut^71^. This was clearly pictured in our OTU network (Fig. 3) where only a few OTUs present in feed were also present in water or gut samples. *Corynebacterium* (OTU 5873) was an exception since it was the predominant OTU in feed but also present in the gut of fish larvae in AS on day 7. *Corynebacteria* are found in a broad variety of habitats such as soil, plants and food products^108^.

**Figure 4:**
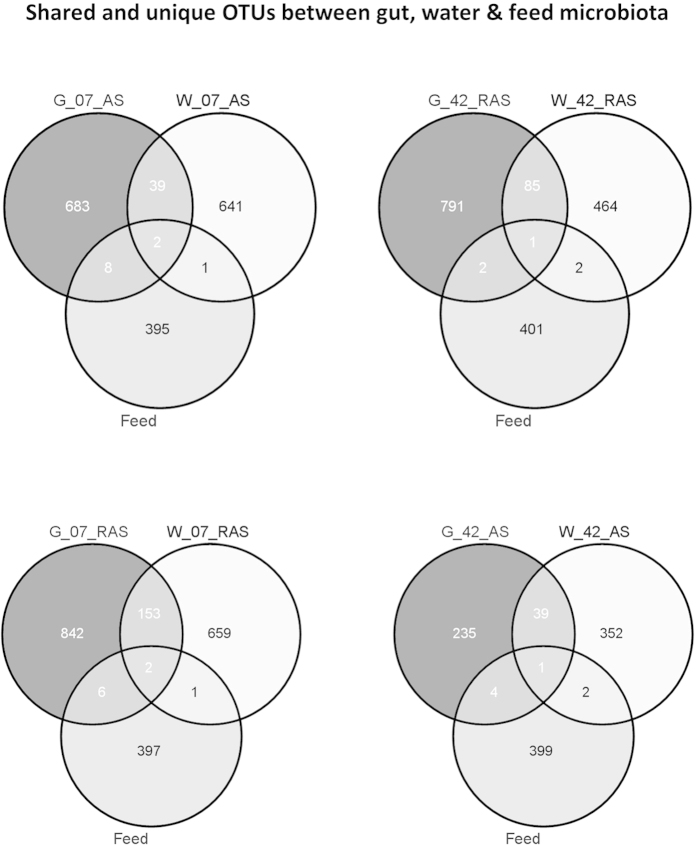
Venn diagrams of bacterial communities between sample types. Number of reads in all samples was normalized to the minimum number of reads found in one of the samples (1719 reads). The Venn diagram illustrates overlap of bacterial OTUs between gut, water and feed samples for two different systems over time. Numbers inside circles indicate the number of unique OTUs in one of the subsets based on absence/presence data from replicate systems. Numbers of unique OTUs indicate those OTUs that were present in both replicate systems and in at least one of the samples within each replicate system. Numbers in the overlapping regions indicate the number of shared OTUs between subsets. AS & RAS: active suspension and recirculating aquaculture systems; 07 and 42: Sampling day 7 and 42 respectively.

The low average similarity and the small number of OTUs shared between gut, water and feed (Table 2, Fig. 4) suggest that the microbial community in the gut is not a simple reflection of the microbes from the environment. In our study, bacterial communities in rearing water influenced those present in the gut; nevertheless they only had a limited effect on the predominant gut species. Factors affecting community development include: suitability of adhesion sites and substrates availability, host physiology, gut anatomy and abiotic components in different sections of the gut, as well as the host’s non-specific and specific immunity^10^,^109^. Thus, the resulting gut microbial community can be very different from the microbiota in water or feed. Sullam *et al*. (2012) stated that fish hindgut microbial communities resemble much more those of mammals, than those present in water or feed. Our study confirms that fish, like other vertebrates, harbor specialized gastrointestinal communities which are a skewed selection of microbes present in water and feed.

## Conclusion

The culture system strongly affected the composition and development of bacterial communities in water. Despite low similarities between gut and water, observed changes in water bacterial communities were highly correlated with changes in the gut. However, there is no clear evidence that bacteria in the water from one system had a larger impact on gut communities than the other. The potential role of the few shared or discriminant species explaining a fraction of the observed similarity was also evaluated. Most of the predominant or shared OTUs in the fish gut have also been found in other fish species, which suggests that these microorganisms fulfil similar roles in the gut of different species. By studying the roles and functionalities of the shared species, novel insights on their niche and ecological adaptability of microbiota in water and gut, and to a lesser extend feed, will be generated. A better understanding of how microbiota in water, feed and gut interact will help improving the design, microbial management and nutrient cycling of fish rearing systems. Future research should focus on disentangling the key processes governing selection and propagation of water and feed microbiota, which ultimately become important species of the microbial community in the adult fish gut.

## Additional Information

**How to cite this article**: Giatsis, C. *et al*. The impact of rearing environment on the development of gut microbiota in tilapia larvae. *Sci. Rep.* **5**, 18206; doi: 10.1038/srep18206 (2015).

## Supplementary Material

Supplementary Information

## Figures and Tables

**Table 1:** ANOVA results with system as main factor and day as repeated measure.

Parameter	System				Day				Syst*Day
	RAS		AS		7		42		
	7	42	7	42	RAS	AS	RAS	AS	
TAN (mg L^−1^)	0.03	0.05	0.06	0.04	0.03	0.06	0.05	0.04	**0.047**
NO2 (mg L^−1^)	0.03		0.11		0.04		0.10		0.303
NO3 (mg L^−1^)	**12.4**	**54.3**	**4.5**	**28.2**	**12.4**	**4.5**	**54.3**	**28.2**	**0.000**
PO4 (mg L^−1^)	**1.46**		**1.04**		**0.37**		**2.13**		0.098
CO2 (mg L^−1^)	**59.1**	**9.4**	**72.9**	**39.0**	**59.1**	**72.9**	**9.4**	**39.0**	**0.003**
Urea (mg L^−1^)	0.02		0.07		0.03		0.06		0.465
T (^o^C)	27.3		27.5		27.2		27.6		0.884
pH	**8.06**	**7.28**	8.15	8.10	8.06	8.15	**7.28**	**8.10**	**0.002**
DO (mg L^−1^)	**7.72**		**8.00**		**8.18**		**7.55**		0.150
Cond (μS cm^−1^)	**402**	**820**	**260**	**456**	**402**	**260**	**820**	**456**	**0.002**

Average water quality parameters calculated for recirculating aquaculture system (RAS) or active suspension (AS) and for day 7 and 42 (n = 4) are given. The right column gives the “System * Day” interaction P values (bold for P < 0.05). Within system or day, bold values indicate significant difference (P < 0.05). TAN: Total ammonia nitrogen; NO2-N: Nitrite nitrogen; NO3-N: Nitrate nitrogen; PO43-P: Phosphate phosphorous; CO2: Carbon dioxide; T: Temperature; DO: Dissolved oxygen; Cond.: Conductivity.

**Table 2:** Percentage average Bray Curtis similarity between gut, water and feed bacterial communities.

% Average Bray Curtis similarity						
	Gut			Water		
	Between replicate AS (AS1, AS2)	Between replicate RAS (Ra, Rb)	Between AS & RAS	Between replicate AS (AS1, AS2)	Between replicate RAS (Ra, Rb)	Between AS & RAS
**Day 7**	68.9 (±7.6)	55.1 (±15.9)	21.6	63.8	59.0	23.9
**Day 42**	59.9 (±18.1)	56.4 (±11.0)	10.8	25.8	60.8	1.8
	**Between day 7 & 42 of gut**			**Between day 7 & 42 of water**		
**AS**	21.6			17.3		
**RAS**	27.2			3.5		
	**Day 7**			**Day 42**		
	Between gut & water	Between gut & feed	Between water & feed	Between gut & water	Between gut & feed	Between water & feed
**AS**	4.1	0.1	0.1	8.5	0.1	0.1
**RAS**	3.9	0.1	0.1	8.1	0.1	0.1

Values are based on SIMPER analysis of square root transformed relative abundance data at the OTU level. Values within brackets indicate the standard deviation of the average gut Bray Curtis similarity within group, as a measure of multivariate within group dispersion. AS & RAS: Active suspension and recirculating aquaculture systems respectively; AS1 & AS2: Replicate active suspension system 1 & 2; Ra & Rb: Replicate recirculating aquaculture system a & b; 7 and 42: Sampling day 7 and 42.

**Table 3:**
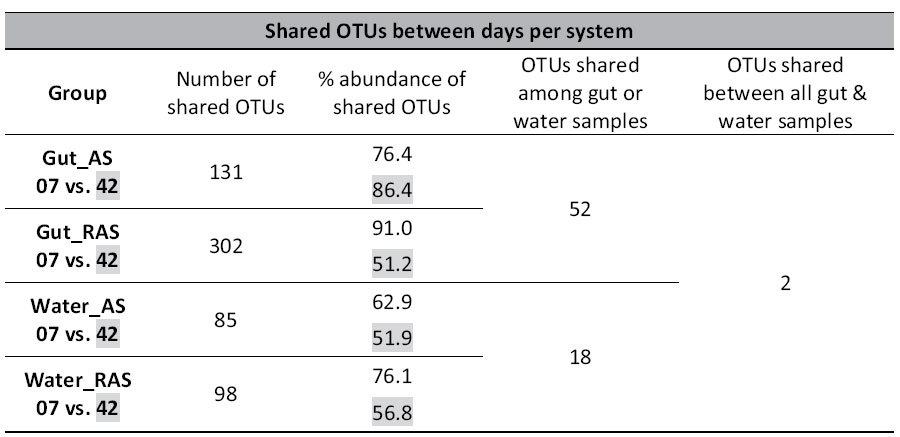
Number of unique and shared OTUs and relative abundance of shared OTUs between days.

Shaded abundance values correspond to shaded sampling group in the first column. Sampling groups are compared between sampling days for each system. OTU: Operational taxonomic unit; AS & RAS: Active suspension and recirculating aquaculture systems; 07 & 42: Sampling days 7 and 42 respectively.

**Table 4:** Number of unique and shared OTUs within and between systems.

Shared OTUs between systems per day			
Group	Total number of OTUs	Number of shared OTUs	Within group % abundance of shared OTUs
Gut_07_AS	732	247	70.7
Gut_07_RAS	1003		71.4
Gut_42_AS	279	90	43.9
Gut_42_RAS	879		50.7
Water_07_AS	683	113	62.5
Water_07_RAS	815		73.0
Water_42_AS	394	40	30.3
Water_42_RAS	552		3.6

Percentages indicate the relative abundance of shared OTUs within each system. Operational taxonomic unit; AS & RAS: Active suspension and recirculating aquaculture systems; 07 & 42: Sampling days 7 and 42 respectively.

**Table 5:**
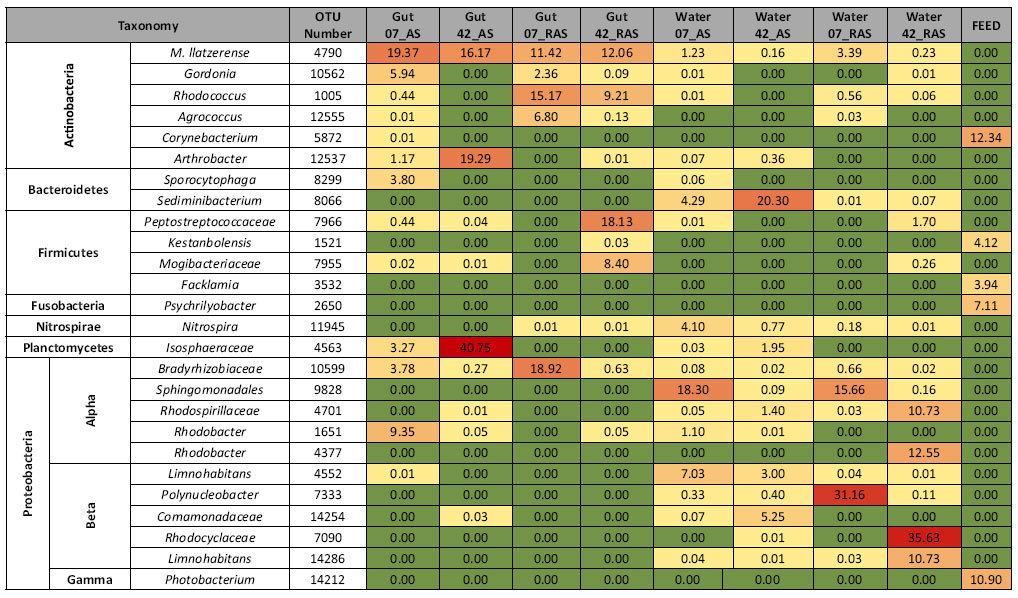
Heatmap of the most predominant gut, water and feed OTUs on day 7 and 42.

Values represent the % average relative abundance of each OTU within each sample group and colour codes are proportional to increasing OTU abundance (from green: lower, to red: higher relative abundance). 07 and D42: sampling day 7 & 42; AS & RAS: Active suspension and recirculating aquaculture systems respectively.

**Table 6:**
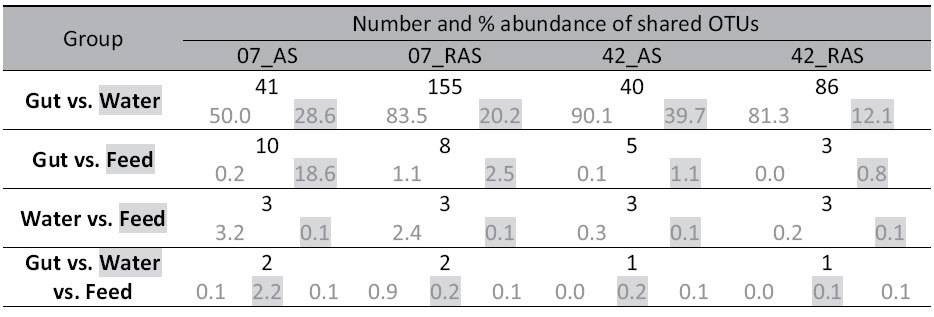
Total number and percentage relative abundance of shared OTUs per system on each sampling day.

Shaded relative abundance values correspond to shaded sampling group in the first column. Sample types are compared within system for each days. OTU: Operational taxonomic unit; AS & RAS: Active suspension and recirculating aquaculture systems; 07 & 42: Sampling days 7 and 42 respectively.
