# Supplementary material for: The impact of rearing environment on the development of gut microbiota in tilapia larvae
Source: Sci Rep. 2015 Dec 11;5:18206. doi: 10.1038/srep18206 (PMC4676014; doi:10.1038/srep18206)
Supplement: Supplementary Information [file srep18206-s1.doc]

**The impact of rearing environment on the development of gut microbiota in tilapia larvae**

Christos Giatsis^1*^, Detmer Sipkema^2^, Hauke Smidt^2^, Hans Heilig^2^, Giulia Benvenuti^3^, Johan Verreth^1^, Marc Verdegem^1^

^1^ Aquaculture and Fisheries Group, Wageningen University, PO Box 338, 6708 WD Wageningen, the Netherlands

^2^ Laboratory of Microbiology, Wageningen University, Dreijenplein 10, 6703 HB Wageningen, the Netherlands

^3^ Bioprocess Engineering, AlgaePARC, Wageningen University, P.O. Box 16, 6700 AA Wageningen, the Netherlands

**Supplementary Table S1. Similarity percentages (SIMPER) indicating the most abundant OTUs contributing to the discrimination between groups, or to the relatedness of samples within a group**. Average similarity: Overall Bray Curtis similarity among samples within the same group; Average dissimilarity: Overall Bray Curtis dissimilarity between samples of the two groups in comparison; Av. Abundance: Average relative abundance of an OTU within a group; Av. Sim.: Contribution of an OTU to the average group similarity; Av. Diss.: Contribution of an OTU to the average dissimilarity between the two groups in comparison; Sim/SD: Ratio of the average contribution of an OTU to the average similarity within the group, divided by the SD (in the case of AS water microbiota, # indicates that no value was calculated due to the lack of replication and estimation of the SD) ; Diss./SD: Ratio of the average contribution of an OTU to the average dissimilarity within the group, divided by the SD (low SD suggests consistency of a discriminant OTU); Contrib%: Percentage contribution of an OTU to the similarity within a group or to the dissimilarity between groups in comparison; Cum.%: Cumulative percentage contribution of OTU’s based on column Contrib%; OTU: Operational taxonomic unit No; AS & RAS: Active suspension and recirculating aquaculture systems respectively; 7 and 42: Sampling day 7 and 42.

| **Type: Gut, Day: 7, System: AS** | | | | | | | | | | | | | | | | | | | | | |
| --- | --- | --- | --- | --- | --- | --- | --- | --- | --- | --- | --- | --- | --- | --- | --- | --- | --- | --- | --- | --- | --- |
| Average similarity: 68.91 | | | | | | | | | | | | | | | | | | | | | |
| Taxonomic ID | Av.Abund | | | | | Av.Sim | | | Sim/SD | | | Contrib% | | | | Cum.% | | | OTU | | |
| Bacteria Actinobacteria Actinobacteria Actinomycetales Mycobacteriaceae Mycobacterium llatzerense | 19.37 | | | | | 13.68 | | | 1.83 | | | 19.85 | | | | 19.85 | | | 4790 | | |
| Bacteria Proteobacteria Alphaproteobacteria Rhodobacterales Rhodobacteraceae Rhodobacter | 9.35 | | | | | 7.01 | | | 3.97 | | | 10.17 | | | | 30.02 | | | 1651 | | |
| Bacteria Actinobacteria Actinobacteria Actinomycetales Gordoniaceae Gordonia | 5.94 | | | | | 4.68 | | | 6.67 | | | 6.79 | | | | 36.81 | | | 10562 | | |
| Bacteria Proteobacteria Alphaproteobacteria Rhizobiales Bradyrhizobiaceae | 3.78 | | | | | 3.22 | | | 4.68 | | | 4.68 | | | | 41.49 | | | 10599 | | |
| Bacteria Bacteroidetes Cytophagia Cytophagales Cytophagaceae Sporocytophaga | 3.8 | | | | | 2.97 | | | 3.87 | | | 4.3 | | | | 45.79 | | | 8299 | | |
| Bacteria Planctomycetes Planctomycetia Pirellulales Pirellulaceae A17 | 3.3 | | | | | 2.79 | | | 4.85 | | | 4.04 | | | | 49.84 | | | 8792 | | |
| Bacteria Planctomycetes Planctomycetia Gemmatales Isosphaeraceae | 3.27 | | | | | 2.46 | | | 2.81 | | | 3.57 | | | | 53.41 | | | 4563 | | |
| Bacteria Proteobacteria Alphaproteobacteria Rhizobiales | 2.54 | | | | | 2.11 | | | 5.9 | | | 3.06 | | | | 56.47 | | | 14302 | | |
| **Type: Gut, Day: 7, System: RAS** | | | | | | | | | | | | | | | | | | | | | |
| Average similarity: 55.06 | | | | | | | | | | | | | | | | | | | | | |
| Taxonomic ID | Av.Abund | | | | | Av.Sim | | | Sim/SD | | | Contrib% | | | | Cum.% | | | OTU | | |
| Bacteria Proteobacteria Alphaproteobacteria Rhizobiales Bradyrhizobiaceae | 18.92 | | | | | 11.59 | | | 1.29 | | | 21.06 | | | | 21.06 | | | 10599 | | |
| Bacteria Actinobacteria Actinobacteria Actinomycetales Nocardiaceae Rhodococcus | 15.17 | | | | | 9.28 | | | 1.37 | | | 16.86 | | | | 37.91 | | | 1005 | | |
| Bacteria Actinobacteria Actinobacteria Actinomycetales Mycobacteriaceae Mycobacterium llatzerense | 11.43 | | | | | 9.02 | | | 3.02 | | | 16.38 | | | | 54.3 | | | 4790 | | |
| Bacteria Actinobacteria Actinobacteria Actinomycetales Microbacteriaceae Agrococcus | 6.8 | | | | | 4.66 | | | 2 | | | 8.47 | | | | 62.77 | | | 12555 | | |
| **Type: Gut, Day: 7, System: AS vs. RAS** | | | | | | | | | | | | | | | | | | | | | |
| Average dissimilarity = 78.38 | | | | | | | | | | | | | | | | | | | | | |
|  | Group AS | | Group RAS | | | | |  | | |  | | |  | | | |  | | | |
| Taxonomic ID | Av.Abund | | Av.Abund | | | | | Av.Diss | | | Diss/SD | | | Contrib% | | | | Cum.% | | | OTU |
| Bacteria Proteobacteria Alphaproteobacteria Rhizobiales Bradyrhizobiaceae | 3.78 | | 18.92 | | | | | 7.58 | | | 1.23 | | | 9.67 | | | | 9.67 | | | 10599 |
| Bacteria Actinobacteria Actinobacteria Actinomycetales Nocardiaceae Rhodococcus | 0.44 | | 15.17 | | | | | 7.36 | | | 1.52 | | | 9.39 | | | | 19.07 | | | 1005 |
| Bacteria Actinobacteria Actinobacteria Actinomycetales Mycobacteriaceae Mycobacterium llatzerense | 19.37 | | 11.43 | | | | | 5.14 | | | 1.35 | | | 6.56 | | | | 25.63 | | | 4790 |
| Bacteria Proteobacteria Alphaproteobacteria Rhodobacterales Rhodobacteraceae Rhodobacter | 9.35 | | 0 | | | | | 4.68 | | | 2.51 | | | 5.96 | | | | 31.59 | | | 1651 |
| Bacteria Actinobacteria Actinobacteria Actinomycetales Microbacteriaceae Agrococcus | 0.01 | | 6.8 | | | | | 3.4 | | | 1.9 | | | 4.33 | | | | 35.93 | | | 12555 |
| Bacteria Actinobacteria Actinobacteria Actinomycetales Gordoniaceae Gordonia | 5.94 | | 2.36 | | | | | 3.27 | | | 1.59 | | | 4.17 | | | | 40.1 | | | 10562 |
| Bacteria Actinobacteria Thermoleophilia Solirubrobacterales | 0 | | 5.79 | | | | | 2.89 | | | 2.72 | | | 3.69 | | | | 43.79 | | | 3934 |
| **Type: Water, Day: 7, System: AS** | | | | | | | | | | | | | | | | | | | | | |
| Average similarity: 63.75 | | | | | | | | | | | | | | | | | | | | | |
| Taxonomic ID | Av.Abund | | | | | Av.Sim | | | Sim/SD | | | Contrib% | | | | Cum.% | | | OTU | | |
| Bacteria Proteobacteria Alphaproteobacteria Sphingomonadales | 18.3 | | | | | 14.75 | | | ####### | | | 23.13 | | | | 23.13 | | | 9828 | | |
| Bacteria Proteobacteria Betaproteobacteria Burkholderiales Comamonadaceae Limnohabitans | 7.03 | | | | | 5.81 | | | ####### | | | 9.12 | | | | 32.25 | | | 4552 | | |
| Bacteria Bacteroidetes Saprospirae Saprospirales Chitinophagaceae Sediminibacterium | 4.29 | | | | | 4.1 | | | ####### | | | 6.43 | | | | 38.67 | | | 8066 | | |
| Bacteria Proteobacteria Alphaproteobacteria Rhodobacterales Rhodobacteraceae Rhodobacter | 5 | | | | | 3.81 | | | ####### | | | 5.98 | | | | 44.66 | | | 2756 | | |
| Bacteria Nitrospirae Nitrospira Nitrospirales Nitrospiraceae Nitrospira | 4.1 | | | | | 2.96 | | | ####### | | | 4.64 | | | | 49.3 | | | 11945 | | |
| **Type: Water, Day: 7, System: RAS** | | | | | | | | | | | | | | | | | | | | | |
| Average similarity: 58.96 | | | | | | | | | | | | | | | | | | | | | |
| Taxonomic ID | | Av.Abund | | | | | Av.Sim | | | Sim/SD | | | Contrib% | | | | Cum.% | | | OTU | |
| Bacteria Proteobacteria Betaproteobacteria Burkholderiales Oxalobacteraceae Polynucleobacter | | 31.16 | | | | | 24.13 | | | 3.1 | | | 40.92 | | | | 40.92 | | | 7333 | |
| Bacteria Proteobacteria Alphaproteobacteria Sphingomonadales | | 15.66 | | | | | 9.97 | | | 1.67 | | | 16.91 | | | | 57.83 | | | 9828 | |
| **Type: Water, Day: 7, System: AS vs. RAS** | | | | | | | | | | | | | | | | | | | | | |
| Average dissimilarity = 76.04 | | | | | | | | | | | | | | | | | | | | | |
|  | | Group AS | | Group RAS | | | |  | | | | | | | | | | | | | |
| Taxonomic ID | | Av.Abund | | Av.Abund | | | | Av.Diss | | | Diss/SD | | | | Contrib% | | | Cum.% | | | OTU |
| Bacteria Proteobacteria Betaproteobacteria Burkholderiales Oxalobacteraceae Polynucleobacter | | 0.33 | | 31.16 | | | | 15.42 | | | 2.94 | | | | 20.28 | | | 20.28 | | | 7333 |
| Bacteria Proteobacteria Alphaproteobacteria Sphingomonadales | | 18.3 | | 15.66 | | | | 3.96 | | | 1.61 | | | | 5.21 | | | 25.49 | | | 9828 |
| Bacteria Proteobacteria Alphaproteobacteria Sphingomonadales | | 7.03 | | 0.04 | | | | 3.49 | | | 5.37 | | | | 4.6 | | | 30.08 | | | 4552 |
| Bacteria Proteobacteria Alphaproteobacteria Sphingomonadales | | 1 | | 5.25 | | | | 2.49 | | | 1.09 | | | | 3.28 | | | 33.36 | | | 10813 |
| Bacteria Proteobacteria Alphaproteobacteria Rhodobacterales Rhodobacteraceae Rhodobacter | | 5 | | 0.11 | | | | 2.44 | | | 3.86 | | | | 3.21 | | | 36.57 | | | 2756 |
| Bacteria Bacteroidetes Saprospirae Saprospirales Chitinophagaceae Sediminibacterium | | 4.29 | | 0.01 | | | | 2.14 | | | 20.93 | | | | 2.81 | | | 39.38 | | | 8066 |
| Bacteria Proteobacteria Alphaproteobacteria Sphingomonadales | | 4.22 | | 0 | | | | 2.11 | | | 2.55 | | | | 2.77 | | | 42.15 | | | 4582 |
| Bacteria Nitrospirae Nitrospira Nitrospirales Nitrospiraceae Nitrospira | | 4.1 | | 0.18 | | | | 1.96 | | | 3.19 | | | | 2.58 | | | 44.73 | | | 11945 |
| Bacteria Actinobacteria Actinobacteria Actinomycetales Mycobacteriaceae Mycobacterium llatzerense | | 1.23 | | 3.39 | | | | 1.4 | | | 1.31 | | | | 1.85 | | | 46.58 | | | 4790 |
| Bacteria Actinobacteria Actinobacteria Actinomycetales Mycobacteriaceae Mycobacterium llatzerense | | 0.01 | | 2.72 | | | | 1.36 | | | 0.94 | | | | 1.79 | | | 48.36 | | | 9738 |
| Bacteria Proteobacteria Betaproteobacteriao Burkholderiales Comamonadaceae | | 2.4 | | 0.29 | | | | 1.17 | | | 1.03 | | | | 1.54 | | | 49.9 | | | 12945 |
| Bacteria Armatimonadetes Armatimonadia Armatimonadales Armatimonadaceae Armatimonas | | 2.09 | | 0 | | | | 1.05 | | | 1.02 | | | | 1.38 | | | 51.28 | | | 7261 |
| Bacteria Proteobacteria Alphaproteobacteria Sphingomonadales Sphingomonadaceae Novosphingobium | | 0.78 | | 2.6 | | | | 0.92 | | | 0.99 | | | | 1.21 | | | 52.49 | | | 6299 |
| **Type: Gut, Day: 42, System: AS** | | | | | | | | | | | | | | | | | | | | | |
| Average similarity: 59.85 | | | | | | | | | | | | | | | | | | | | | |
| Taxonomic ID | | Av.Abund | | | | | Av.Sim | | | Sim/SD | | | Contrib% | | | | Cum.% | | | OTU | |
| Bacteria Planctomycetes Planctomycetia Gemmatales Isosphaeraceae | | 40.76 | | | | | 27.08 | | | 1.56 | | | 45.25 | | | | 45.25 | | | 4563 | |
| Bacteria Actinobacteria Actinobacteria Actinomycetales Micrococcaceae Arthrobacter | | 19.29 | | | | | 14.57 | | | 3.77 | | | 24.34 | | | | 69.59 | | | 12537 | |
| Bacteria Actinobacteria Actinobacteria Actinomycetales Mycobacteriaceae Mycobacterium llatzerense | | 16.17 | | | | | 8.74 | | | 0.97 | | | 14.6 | | | | 84.19 | | | 4790 | |
| **Type: Gut, Day: 42, System: RAS** | | | | | | | | | | | | | | | | | | | | | |
| Average similarity: 56.35 | | | | | | | | | | | | | | | | | | | | | |
| Taxonomic ID | | Av.Abund | | | | | Av.Sim | | | Sim/SD | | | Contrib% | | | | Cum.% | | | OTU | |
| Bacteria Firmicutes Clostridia Clostridiales Peptostreptococcaceae | | 18.13 | | | | | 13.02 | | | 2.8 | | | 23.11 | | | | 23.11 | | | 7966 | |
| Bacteria Actinobacteria Actinobacteria Actinomycetales Mycobacteriaceae Mycobacterium llatzerense | | 12.06 | | | | | 9.44 | | | 2.99 | | | 16.75 | | | | 39.86 | | | 4790 | |
| Bacteria Firmicutes Clostridia Clostridiales [Mogibacteriaceae] | | 8.4 | | | | | 6.16 | | | 2.21 | | | 10.93 | | | | 50.79 | | | 7955 | |
| Bacteria Actinobacteria Actinobacteria Actinomycetales Nocardiaceae Rhodococcus | | 9.21 | | | | | 5.74 | | | 1.73 | | | 10.18 | | | | 60.96 | | | 1005 | |
| Bacteria Fusobacteria Fusobacteriia Fusobacteriales Fusobacteriaceae Cetobacterium somerae | | 11.73 | | | | | 4.31 | | | 0.75 | | | 7.64 | | | | 68.61 | | | 2787 | |
| **Type: Gut, Day: 42, System: AS vs. RAS** | | | | | | | | | | | | | | | | | | | | | |
| Average dissimilarity = 89.22 | | | | | | | | | | | | | | | | | | | | | |
|  | | Group AS | | Group RAS | | | |  | | | | | | | | | | | | | |
| Taxonomic ID | | Av.Abund | | Av.Abund | | | | Av.Diss | | | Diss/SD | | | | Contrib% | | | Cum.% | | | OTU |
| Bacteria Planctomycetes Planctomycetia Gemmatales Isosphaeraceae | | 40.76 | | 0 | | | | 20.38 | | | 1.91 | | | | 22.84 | | | 22.84 | | | 4563 |
| Bacteria Actinobacteria Actinobacteria Actinomycetales Micrococcaceae Arthrobacter | | 19.29 | | 0.01 | | | | 9.64 | | | 2.57 | | | | 10.8 | | | 33.64 | | | 12537 |
| Bacteria Firmicutes Clostridia Clostridiales Peptostreptococcaceae | | 0.04 | | 18.13 | | | | 9.04 | | | 2.01 | | | | 10.14 | | | 43.78 | | | 7966 |
| Bacteria Fusobacteria Fusobacteriia Fusobacteriales Fusobacteriaceae Cetobacterium somerae | | 0 | | 11.73 | | | | 5.86 | | | 0.85 | | | | 6.57 | | | 50.35 | | | 2787 |
| Bacteria Actinobacteria Actinobacteria Actinomycetales Mycobacteriaceae Mycobacterium llatzerense | | 16.17 | | 12.06 | | | | 5.78 | | | 1.87 | | | | 6.48 | | | 56.83 | | | 4790 |
| Bacteria Actinobacteria Actinobacteria Actinomycetales Nocardiaceae Rhodococcus | | 0 | | 9.21 | | | | 4.6 | | | 1.56 | | | | 5.16 | | | 61.99 | | | 1005 |
| Bacteria Firmicutes Clostridia Clostridiales Mogibacteriaceae | | 0.01 | | 8.4 | | | | 4.2 | | | 2.3 | | | | 4.7 | | | 66.69 | | | 7955 |
| Bacteria Proteobacteria Gammaproteobacteria Legionellales Legionellaceae Tatlockia | | 6.02 | | 0 | | | | 3.01 | | | 0.78 | | | | 3.37 | | | 70.07 | | | 2401 |
| Bacteria Proteobacteria Gammaproteobacteria Enterobacteriales Enterobacteriaceae Plesiomonas shigelloides | | 1.77 | | 2.63 | | | | 1.53 | | | 1.07 | | | | 1.72 | | | 71.79 | | | 12852 |
| Bacteria Actinobacteria Actinobacteria Actinomycetales Mycobacteriaceae Mycobacterium celatum | | 0.01 | | 3.04 | | | | 1.51 | | | 0.99 | | | | 1.7 | | | 73.48 | | | 12238 |
| Bacteria Actinobacteria Thermoleophilia Solirubrobacterales | | 0 | | 2.73 | | | | 1.37 | | | 1.15 | | | | 1.53 | | | 75.01 | | | 3934 |
| **Type: Water, Day: 42, System: AS** | | | | | | | | | | | | | | | | | | | | | |
| Average similarity: 25.82 | | | | | | | | | | | | | | | | | | | | | |
| Taxonomic ID | | Av.Abund | | | | | Av.Sim | | | Sim/SD | | | Contrib% | | | | Cum.% | | | OTU | |
| Bacteria Bacteroidetes Saprospirae Saprospirales Chitinophagaceae Sediminibacterium | | 20.31 | | | | | 11.43 | | | ####### | | | 44.28 | | | | 44.28 | | | 8066 | |
| Bacteria Proteobacteria Betaproteobacteria Burkholderiales Comamonadaceae | | 5.25 | | | | | 2.9 | | | ####### | | | 11.24 | | | | 55.52 | | | 14254 | |
| Bacteria Proteobacteria Alphaproteobacteria Rhodospirillales Rhodospirillaceae | | 1.4 | | | | | 1.19 | | | ####### | | | 4.6 | | | | 60.12 | | | 4701 | |
| Bacteria Bacteroidetes Sphingobacteriia Sphingobacteriales | | 7.02 | | | | | 0.93 | | | ####### | | | 3.58 | | | | 63.7 | | | 4686 | |
| **Type: Water, Day: 42, System: RAS** | | | | | | | | | | | | | | | | | | | | | |
| Average similarity: 60.79 | | | | | | | | | | | | | | | | | | | | | |
| Taxonomic ID | | Av.Abund | | | | | Av.Sim | | | Sim/SD | | | Contrib% | | | | Cum.% | | | OTU | |
| Bacteria Proteobacteria Betaproteobacteria Rhodocyclales Rhodocyclaceae | | 35.64 | | | | | 30.69 | | | 3.97 | | | 50.48 | | | | 50.48 | | | 7090 | |
| Bacteria Proteobacteria Betaproteobacteria Burkholderiales Comamonadaceae Limnohabitans | | 10.73 | | | | | 6.8 | | | 4.31 | | | 11.18 | | | | 61.66 | | | 14286 | |
| Bacteria Proteobacteria Alphaproteobacteria Rhodobacterales Rhodobacteraceae Rhodobacter | | 12.56 | | | | | 3.32 | | | 0.41 | | | 5.47 | | | | 67.13 | | | 4377 | |
| **Type: Water, Day: 42, System: AS vs. RAS** | | | | | | | | | | | | | | | | | | | | | |
| Average dissimilarity = 98.24 | | | | | | | | | | | | | | | | | | | | | |
|  | | Group AS | | | Group RAS | | |  | | |  | | | |  | | |  | | | |
| Taxonomic ID | | Av.Abund | | | Av.Abund | | | Av.Diss | | | Diss/SD | | | | Contrib% | | | Cum.% | | | OTU |
| Bacteria Proteobacteria Betaproteobacteria Rhodocyclales Rhodocyclaceae | | 0.01 | | | 35.64 | | | 17.82 | | | 4.63 | | | | 18.14 | | | 18.14 | | | 7090 |
| Bacteria Bacteroidetes Saprospirae Saprospirales Chitinophagaceae Sediminibacterium | | 20.31 | | | 0.07 | | | 10.12 | | | 2.13 | | | | 10.3 | | | 28.43 | | | 8066 |
| Bacteria Armatimonadetes Fimbriimonadia Fimbriimonadales Fimbriimonadaceae Fimbriimonas | | 14.54 | | | 0 | | | 7.27 | | | 0.94 | | | | 7.4 | | | 35.84 | | | 5688 |
| Bacteria Proteobacteria Alphaproteobacteria Rhodobacterales Rhodobacteraceae Rhodobacter | | 0 | | | 12.56 | | | 6.28 | | | 0.9 | | | | 6.39 | | | 42.23 | | | 4377 |
| Bacteria Proteobacteria Betaproteobacteria Burkholderiales Comamonadaceae Limnohabitans | | 0.01 | | | 10.73 | | | 5.36 | | | 1.71 | | | | 5.46 | | | 47.68 | | | 14286 |
| Bacteria Armatimonadetes Armatimonadia Armatimonadales Armatimonadaceae Armatimonas | | 8.11 | | | 0 | | | 4.05 | | | 0.94 | | | | 4.13 | | | 51.81 | | | 7261 |
| Bacteria Bacteroidetes Sphingobacteriia Sphingobacteriales | | 7.02 | | | 0 | | | 3.51 | | | 1.08 | | | | 3.57 | | | 55.39 | | | 4686 |
| Bacteria Proteobacteria Betaproteobacteria Burkholderiales Comamonadaceae | | 5.25 | | | 0 | | | 2.63 | | | 2.09 | | | | 2.67 | | | 58.06 | | | 14254 |
| Bacteria Fusobacteria Fusobacteriia Fusobacteriales Fusobacteriaceae Cetobacterium somerae | | 0 | | | 5.02 | | | 2.51 | | | 1.58 | | | | 2.55 | | | 60.61 | | | 2787 |
| Bacteria Actinobacteria Actinobacteria Actinomycetales ACK-M1 | | 0 | | | 4.96 | | | 2.48 | | | 0.9 | | | | 2.52 | | | 63.14 | | | 5270 |
| **Type: Feed** | | | | | | | | | | | | | | | | | | | | | |
| Average similarity: 59.36 | | | | | | | | | | | | | | | | | | | | | |
| Taxonomic ID | Av.Abund | | | | | Av.Sim | | | Sim/SD | | | Contrib% | | | | Cum.% | | | OTU | | |
| Bacteria Proteobacteria Gammaproteobacteria Vibrionales Vibrionaceae Photobacterium | 10.9 | | | | | 9.94 | | | ####### | | | 16.75 | | | | 16.75 | | | 14212 | | |
| Bacteria Fusobacteria Fusobacteriia Fusobacteriales Fusobacteriaceae Psychrilyobacter | 7.11 | | | | | 6.78 | | | ####### | | | 11.42 | | | | 28.16 | | | 2650 | | |
| Bacteria Firmicutes Bacilli Lactobacillales Aerococcaceae Facklamia | 3.94 | | | | | 3.83 | | | ####### | | | 6.46 | | | | 34.62 | | | 3532 | | |
| Bacteria Firmicutes Bacilli Bacillales Bacillaceae Anoxybacillus kestanbolensis | 4.12 | | | | | 3.78 | | | ####### | | | 6.37 | | | | 40.99 | | | 1521 | | |
| Bacteria Firmicutes Bacilli Bacillales Planococcaceae Sporosarcina | 8.06 | | | | | 3.2 | | | ####### | | | 5.39 | | | | 46.38 | | | 3747 | | |
| Bacteria Actinobacteria Actinobacteria Actinomycetales Corynebacteriaceae Corynebacterium | 12.34 | | | | | 2.94 | | | ####### | | | 4.95 | | | | 51.32 | | | 5872 | | |

| **Type: Gut, System: AS, Day: 7 vs. 42** | | | | | | | |
| --- | --- | --- | --- | --- | --- | --- | --- |
| Average dissimilarity = 78.39 | | | | | | | |
|  | Group 7 | Group 42 |  |  |  |  |  |
| Species | Av.Abund | Av.Abund | Av.Diss | Diss/SD | Contrib% | Cum.% | OTU |
| Bacteria Planctomycetes Planctomycetia Gemmatales Isosphaeraceae | 3.27 | 40.76 | 18.74 | 1.75 | 23.91 | 23.91 | 4563 |
| Bacteria Actinobacteria Actinobacteria Actinomycetales Micrococcaceae Arthrobacter | 1.17 | 19.29 | 9.06 | 2.4 | 11.55 | 35.46 | 12537 |
| Bacteria Actinobacteria Actinobacteria Actinomycetales Mycobacteriaceae Mycobacterium llatzerense | 19.37 | 16.17 | 6.14 | 1.35 | 7.84 | 43.3 | 4790 |
| Bacteria Proteobacteria Alphaproteobacteria Rhodobacterales Rhodobacteraceae Rhodobacter | 9.35 | 0.05 | 4.65 | 2.48 | 5.93 | 49.23 | 1651 |
| Bacteria Proteobacteria Gammaproteobacteria Legionellales Legionellaceae Tatlockia | 0 | 6.02 | 3.01 | 0.78 | 3.84 | 53.07 | 2401 |
| Bacteria Actinobacteria Actinobacteria Actinomycetales Gordoniaceae Gordonia | 5.94 | 0 | 2.97 | 2.66 | 3.79 | 56.86 | 10562 |
| **Type: Gut, System: RAS, Day: 7 vs. 42** | | | | | | | |
| Average dissimilarity = 72.78 | | | | | | | |
|  | Group 7 | Group 42 |  |  |  |  |  |
| Species | Av.Abund | Av.Abund | Av.Diss | Diss/SD | Contrib% | Cum.% | OTU |
| Bacteria Proteobacteria Alphaproteobacteria Rhizobiales Bradyrhizobiaceae | 18.92 | 0.63 | 9.14 | 1.48 | 12.56 | 12.56 | 10599 |
| Bacteria Firmicutes Clostridia Clostridiales Peptostreptococcaceae | 0 | 18.13 | 9.07 | 2.02 | 12.45 | 25.01 | 7966 |
| Bacteria Fusobacteria Fusobacteriia Fusobacteriales Fusobacteriaceae Cetobacterium somerae | 0 | 11.73 | 5.86 | 0.86 | 8.05 | 33.07 | 2787 |
| Bacteria Actinobacteria Actinobacteria Actinomycetales Nocardiaceae Rhodococcus | 15.17 | 9.21 | 5.03 | 1.28 | 6.91 | 39.98 | 1005 |
| Bacteria Firmicutes Clostridia Clostridiales Mogibacteriaceae | 0 | 8.4 | 4.2 | 2.31 | 5.77 | 45.75 | 7955 |
| Bacteria Actinobacteria Actinobacteria Actinomycetales Microbacteriaceae Agrococcus | 6.8 | 0.13 | 3.33 | 1.87 | 4.58 | 50.33 | 12555 |

| **Type: Water, System: AS, Day: 7 vs. 42** | | | | | | | |
| --- | --- | --- | --- | --- | --- | --- | --- |
| Average dissimilarity = 83.74 | | | | | | | |
|  | Group 7 | Group 42 |  |  |  |  |  |
| Species | Av.Abund | Av.Abund | Av.Diss | Diss/SD | Contrib% | Cum.% | OTU |
| Bacteria Proteobacteria Alphaproteobacteria Sphingomonadales | 18.3 | 0.09 | 9.11 | 4.43 | 10.88 | 10.88 | 9828 |
| Bacteria Bacteroidetes Saprospirae Saprospirales Chitinophagaceae Sediminibacterium | 4.29 | 20.31 | 8.01 | 1.56 | 9.56 | 20.44 | 8066 |
| Bacteria Armatimonadetes Fimbriimonadia Fimbriimonadales Fimbriimonadaceae Fimbriimonas | 0 | 14.54 | 7.27 | 0.87 | 8.68 | 29.12 | 5688 |
| Bacteria Armatimonadetes Armatimonadia Armatimonadales Armatimonadaceae Armatimonas | 2.09 | 8.11 | 4.05 | 1.11 | 4.84 | 33.97 | 7261 |
| Bacteria Bacteroidetes Sphingobacteriia Sphingobacteriales | 0.01 | 7.02 | 3.51 | 1 | 4.19 | 38.15 | 4686 |
| Bacteria Proteobacteria Betaproteobacteria Burkholderiales Comamonadaceae | 0.07 | 5.25 | 2.59 | 1.91 | 3.09 | 41.25 | 14254 |
| **Type: Water, System: RAS, Day: 7 vs. 42** | | | | | | | |
| Average dissimilarity = 96.46 | | | | | | | |
|  | Group 7 | Group 42 |  |  |  |  |  |
| Species | Av.Abund | Av.Abund | Av.Diss | Diss/SD | Contrib% | Cum.% | OTU |
| Bacteria Proteobacteria Betaproteobacteria Rhodocyclales Rhodocyclaceae | 0 | 35.64 | 17.82 | 4.79 | 18.47 | 18.47 | 7090 |
| Bacteria Proteobacteria Betaproteobacteria Burkholderiales Oxalobacteraceae Polynucleobacter | 31.16 | 0.11 | 15.52 | 3.06 | 16.09 | 34.57 | 7333 |
| Bacteria Proteobacteria Alphaproteobacteria Sphingomonadales | 15.66 | 0.16 | 7.75 | 1.87 | 8.04 | 42.6 | 9828 |
| Bacteria Proteobacteria Alphaproteobacteria Rhodobacterales Rhodobacteraceae Rhodobacter | 0 | 12.56 | 6.28 | 0.93 | 6.51 | 49.11 | 4377 |
| Bacteria Proteobacteria Betaproteobacteria Burkholderiales Comamonadaceae Limnohabitans | 0.03 | 10.73 | 5.35 | 1.76 | 5.55 | 54.66 | 14286 |

**Supplementary Table S2. Average alpha-diversity indices of gut, water and feed bacterial communities.** Number of obtained sequences was standardized among samples to the minimum number of reads obtained in one of the samples (n = 1719). S: Richness (observed OTUs); J': Pielou’s evenness (J’=H’/log(S)); H’: Shannon index (H’=-SUM(P_i_*ln(P_i_))), based on the OTU level. AS & RAS: Active suspension and recirculating aquaculture systems; 07 & 42: Sampling days 7 and 42 respectively.

| Sampling Group | S | J' | H'(ln) |
| --- | --- | --- | --- |
| **Gut_07_AS** | 732 | 0.626 | 4.132 |
| **Gut_07_RAS** | 1003 | 0.532 | 3.674 |
| **Gut_42_AS** | 279 | 0.395 | 2.225 |
| **Gut_42_RAS** | 879 | 0.519 | 3.521 |
| **Water_07_AS** | 683 | 0.690 | 4.504 |
| **Water_07_RAS** | 815 | 0.540 | 3.618 |
| **Water_42_AS** | 394 | 0.601 | 3.594 |
| **Water_42_RAS** | 552 | 0.494 | 3.118 |
| **Feed** | 406 | 0.666 | 3.997 |

**Supplementary Table S3. RELATE analysis testing for matched resemblance matrices based on Spearman’s rank correlation coefficient**. Water and gut microbiota data sets were square root transformed and resemblance matrices were based on Bray Curtis similarity among samples. Rho statistic indicates the degree of “relatedness” between the two matrices with highly related matrices having Rho statistic closer to one. BCs: Bacterial communities.

| **Resemblance matrices** | **Water BCs**  **Vs.**  **Gut BCs** |
| --- | --- |
| **Rank correlation method** | Spearman’s p |
| **Sample statistic (Rho)** | 0.807 |
| **Significance level of sample statistic** | 0.0001 |
| **Number of permutations** | 9999 |
| **Number of permuted statistics greater than or equal to Rho** | 0 |

**Supplementary Table S4. List of the most predominant gut, water and feed OTUs on day 7 and 42 as presented on the heatmap Table 3**. Column “Processes” and “Environment” provide information about the major functions and processes as well as the environments in which the most predominant OTUs were encountered as predicted based on a literature review (column “References”).

| Ta**xonomy** | **OTU number** | **Processes** | **Environment** | **References** |
| --- | --- | --- | --- | --- |
| Actinobacteria Actinomycetales Mycobacteriaceae *Mycobacterium llatzerense* | 4790 | Facultative autotropic, aerobic, hydrogen oxidation | Haemodialysis water; tap water production plant and distribution system | 1,2 |
| Proteobacteria Alphaproteobacteria Rhodobacterales Rhodobacteraceae *Rhodobacter* | 1651 | Non-sulphur purple bacteria, photosynthesis, lithotrophs, aerobic and anaerobic respiration, N_2_ fixation | Hypersaline, saline and freshwater | 3–8 |
| Actinobacteria Actinobacteria Actinomycetales Gordoniaceae *Gordonia* | 10562 | High GC-content, Gram+ bacteria, degrade many xenobiotics, potentially pathogenic. | Soil and mangrove rhizospheres, waste water treatment plants, diseased humans, hydrocarbon polluted soil, oil wells | 9 |
| Proteobacteria Alphaproteobacteria Rhizobiales Bradyrhizobiaceae | 10599 | Contains 10 genera, rhizobium associated and animal associated (e.g. cat scratch disease). Many free living in marine or fresh water, some with N_2_-fixation. | Sewage treatment plants, municipal waste waters, drinking water distribution. In gut of sea bass and stomach of yellow catfish. | 8,10–13 |
| Actinobacteria Actinobacteria Actinomycetales Nocardiaceae *Rhodococcus* | 1005 | Chemoheterotrophic, some members are facultative chemo-lithoauthrophs. Many processes. Some species used as immunostimulants, others for production of digestive enzymes. | Wide distribution, including soil, water and in cells. Reported in sole, red rock fish, Norwegian mackerel, USA smelt, rainbow trout and shrimp gut | 14–20 |
| Proteobacteria Alphaproteobacteria Sphingomonadales | 9828 | No info about functional role. Sphingolipids in outer membrane of cell wall. | Drinking water systems, groundwater treatment biofilms. | 10,21–24 |
| Proteobacteria Betaproteobacteria Burkholderiales Comamonadaceae *Limnohabitans* | 4552 | Aerobic, facultative anaerobic, chemo-organotrophic. Affinity for substrates with organic acids, monosaccharides and amino acids. | Bacteria found in water column of freshwaters. | 25–30 |
| Bacteroidetes Saprospirae  Saprospirales Chitinophagaceae *Sediminibacterium* | 8066 | Versatile in types of substrates that are degraded. Aerobic or facultative anaerobic. | Lakes, freshwater reservoirs, activated sludge reactors, stream biofilms, soil | 31–36 |
| Proteobacteria Betaproteobacteria Burkholderiales Oxalobacteraceae *Polynucleobacter* | 7333 | Assimilatory reduction of NO_3_, but no nitrification or denitrification. Heterotrophic bacteria, all amino acid pathways. | Some are obligate endosymbionts of ciliates belonging to the genus *Euplotes.* Free living heterotrophs, providing up to 60% of total bacteria numbers in freshwater. | 37–41 |
| Planctomycetes Planctomycetia Gemmatales Isosphaeraceae | 4563 | Functional role of gut microbiota unknown | Peat soils, aerosols, soil samples | 42–44 |
| Actinobacteria Actinobacteria Actinomycetales Micrococcaceae *Arthrobacter* | 12537 | Gram positive obligate aerobes. Probiotics, increasing disease resistance and survival | Common in soils. In gut of grass carp, Atlantic cod, rainbow trout, Atlantic salmon, cobia and halibut. Used in bio-remediation of soils. | 45–52 |
| Firmicutes Clostridia Clostridiales Peptostreptococcaceae | 7966 | Gram positive obligate anaerobes, fermentation or organic matter. | Considered part of intestinal microbiota of Atlantic salmon. Reported in gut of yellow catfish and common carp, in waste water treatment and active sludge bioreactors. | 53–59 |
| Firmicutes Clostridia Clostridiales Mogibacteriaceae | 7955 | No information found | In human and mice gut | 60,61 |
| Proteobacteria Betaproteobacteria Burkholderiales Comamonadaceae | 14254 | PHA degradation, denitrification. | In waste water and waste water treatment facilities. | 62–69 |
| Proteobacteria Alphaproteobacteria Rhodospirillales Rhodospirillaceae | 4701 | Family includes chemo-organotrophs, chemo-lithotrophs and facultative photo-heterotrophs. | Freshwater, brackish and marine sediments, activated sludge biomass, biofilms. Contribute to nitrogen metabolism. | 11,69–72 |
| Proteobacteria Betaproteobacteria Rhodocyclales Rhodocyclaceae | 7090 | Gram negative bacteria, containing mainly aerobic or denitrifying bacteria. | Many species prefer oligotrophic conditions, Contribute to biological remediation and waste water treatment. Found in activated sludge. | 57,73–77 |
| Proteobacteria Betaproteobacteria Burkholderiales Comamonadaceae *Limnohabitans* | 14286 | Metabolic flexible, associated to algal-derived organic substances and exudates. Aerobic, facultative anaerobic or chemo-organotrophic. | Freshwater habitats of rivers, lakes and reservoirs. | 25–30 |
| Proteobacteria Alphaproteobacteria Rhodobacterales Rhodobacteraceae *Rhodobacter* | 4377 | Metabolic flexible, including photosynthesis, lithotrophy, and aerobic and anaerobic respiration. Some members fix N_2_. Synnthesize chlorophylls, heme and vitamin B12. | Widely distributed in fresh water, marine and hyper saline environments. Survive in light and dark conditions. | 3–8 |
| Proteobacteria Gammaproteobacteria Vibrionales Vibrionaceae *Photobacterium* | 14212 | Chemo-organotrophs, luminescent gram-negative bacteria, requiring sodium. No endospore formation. | Common in marine environments, free living or in symbiosis with fish. Some species linked to light organs pathogens in fish. Found in gut of Norwegian lobster, red snapper, Atlantic mackerel. | 78–85 |
| Fusobacteria Fusobacteriia Fusobacteriales Fusobacteriaceae *Psychrilyobacter* | 2650 | Fermenting bacteria, producing hydrogen and acetate. Degradation of cyanobacterial biomass in anoxic tidal flat sediments. | Found on Pacific oyster and in gut of mud crab. Associated with biofilm formation in seawater. | 86–91 |
| Firmicutes Bacilli Lactobacillales Aerococcaceae Facklamia | 3532 | Gram-positive, lactic acid bacteria. | Human mucosae, lactating cows, milk products. | 92–98 |
| Bacteria Bacteroidetes Cytophagia Cytophagales Cytophagaceae Sporocytophaga | 8299 | Cellulolytic properties of the *Sporocytophaga* has been reported. | Reported in lake water and soil. In fish, were identified as part of the skin microbiota of brook charr (*Salvelinus fontinalis*) in the gut of rainbow trout (*Oncorhynchus mykiss*), and in the gut of the detritivorous catfish (*P. nigrolineatus*) | 16,99–102 |
| Bacteria Actinobacteria Actinobacteria Actinomycetales Microbacteriaceae Agrococcus | 12555 | Demonstrated to exhibit properties for growth inhibition of *A. salmonicida*, *V. anguillarum*, and *C. maltaromaticum* based on an in vitro test. | Have been reported as predominant autochthonous bacteria in the gut of Atlantic salmon (*Salmo salar L.*). Isolated from skin, gills, intestinal digesta and adherent bacteria of the yellow grouper (*Epinephelus awoora*). | 103,104 |
| Bacteria Nitrospirae Nitrospira Nitrospirales Nitrospiraceae Nitrospira | 11945 | Aerobic lithoautotrophic nitrite-oxidizing It is part of a nitrification process which is important in the biogeochemical nitrogen cycle. | Commonly found in aquaculture systems (filter materials in biofilters of freshwater, brackish and marine systems). Also found in freshwater activated sludge particles, freshwater aquaria biofilms, aquaculture wastewater treatment plants ^105^ but also in rivers, lakes and estuarine sediments. | 106–115 |
| Bacteria Firmicutes Bacilli Bacillales Bacillaceae Anoxybacillus kestanbolensis | 1521 | Facultative anaerobes, Gram-positive, motile, spore-forming rods with spherical endospores. Able to hydrolyze starch and utilize D-mannitol, D-glucose, D-fructose, maltose, D-mannose, D-raffinose and D-sucrose. | Reported in subsurface aquifer sediment and hot spring waters. | 116–118 |
| Bacteria Actinobacteria Actinobacteria Actinomycetales Corynebacteriaceae Corynebacterium | 5872 | Aerobic and facultative anaerobic, gram+, non-sporing, non-motile, chemoorganotrophic bacteria. Many species of show a fermentative metabolism and versatility on growth on carbohydrates, or co-metabolize glucose, fructose, lactate, and pyruvate. | Reported in soil, plants and food products. | 119–121 |

**Supplementary Figure S1. Draftsman plots for the untransformed water quality parameters**. Pairwise plots provide indication of data skewness or multi-colinearity among parameters for data transformation, or exclusion of parameters from the distance-based linear models analysis. TAN: Total ammonia nitrogen; NO_2_^-^-N: Nitrite nitrogen; NO_3_^-^-N: Nitrate nitrogen; PO_4_^3-^-P: Phosphate phosphorous; CO_2_: Carbon dioxide; T: Temperature; DO: Dissolved oxygen; Cond.: Conductivity.

**
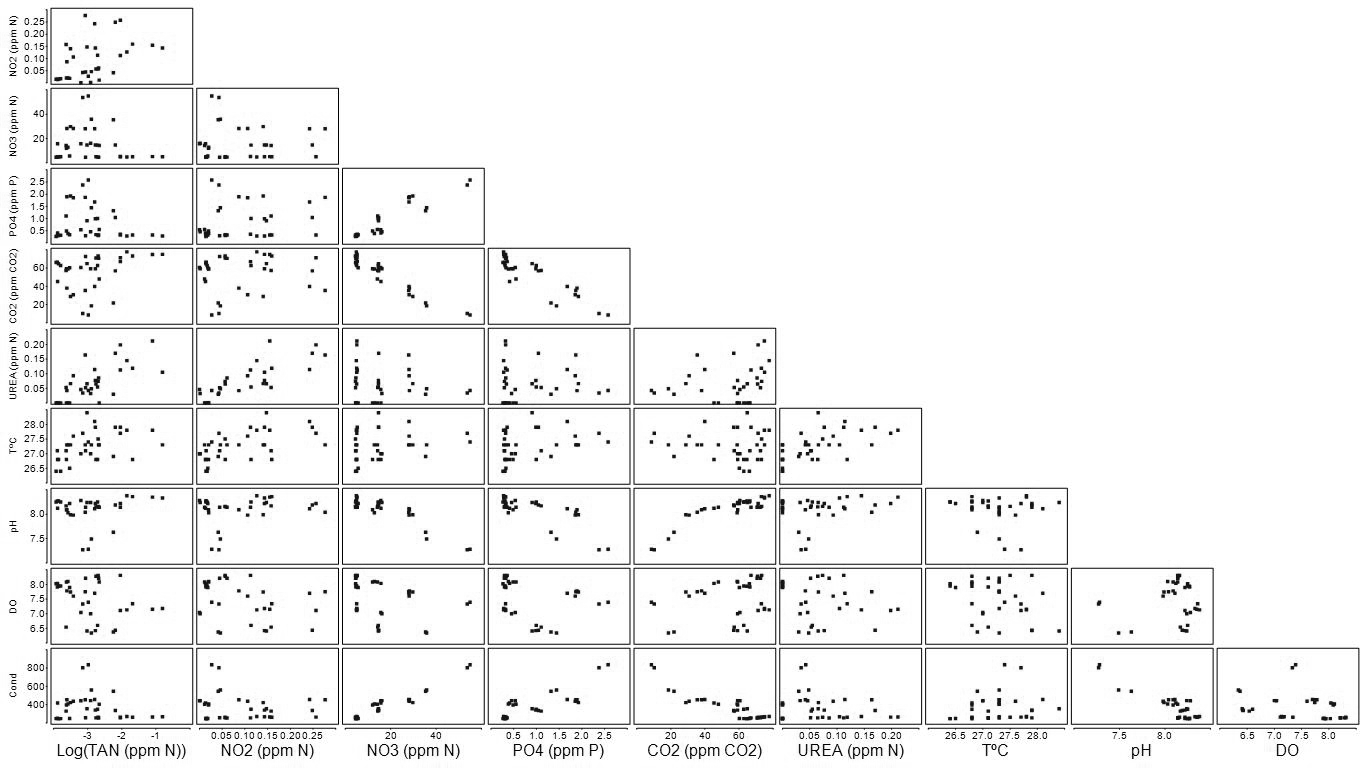
**

**Supplementary Figure S2. Dominance plots of gut and water OTUs from the two experimental days.** Plots are based on average relative Bray Curtis OTU abundance from each system. The x-axis shows the log scale of the OTUs ranks in increasing order and the y-axis consists of the cumulative relative abundance of each OTU in each sample group. 07 and 42: sampling day 7 & 42; AS & RAS: Active suspension and recirculating aquaculture systems respectively.


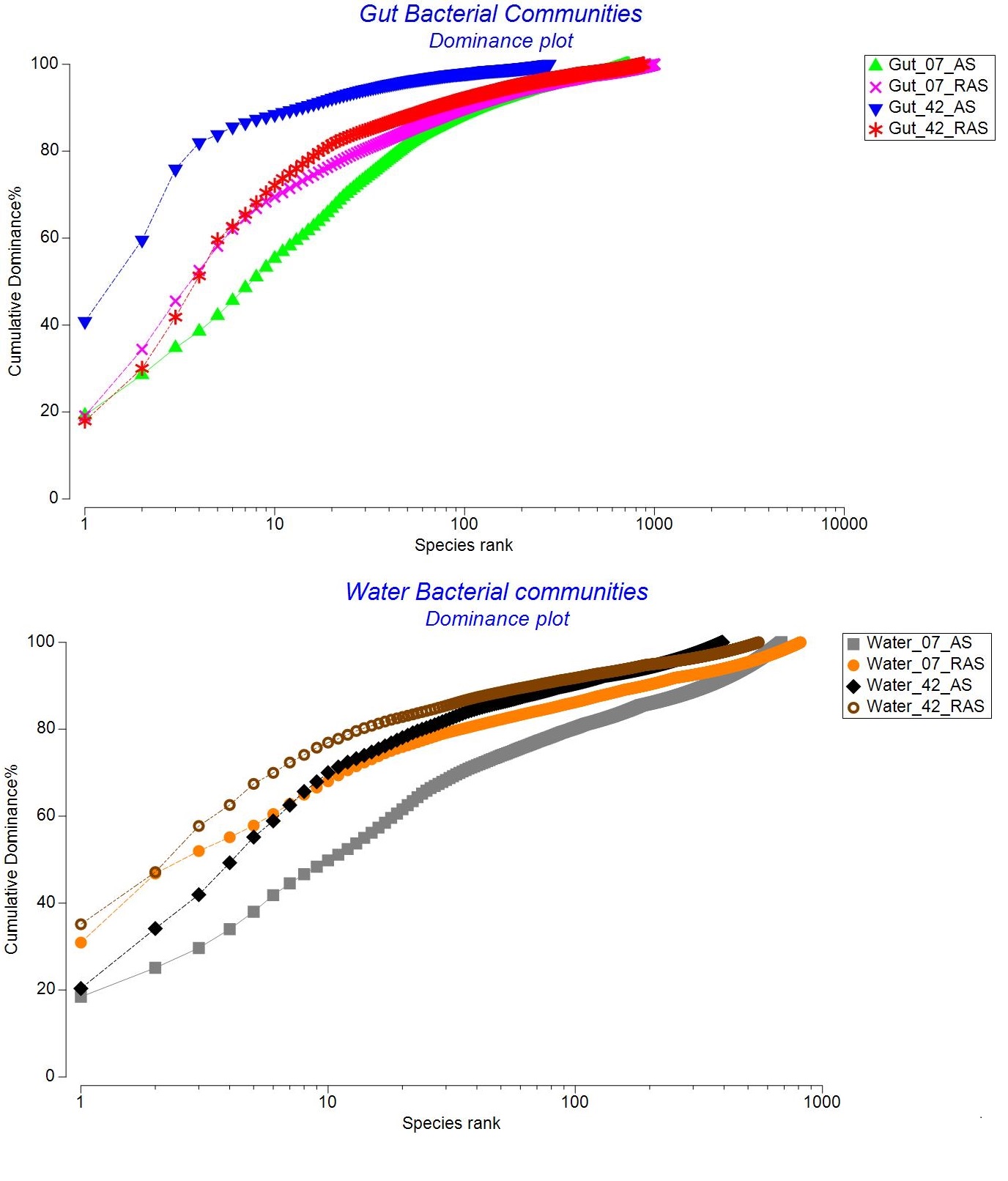


**Figure S3.** **Bayesian phylogram of selected 16S rRNA gene sequences from this study (in bold) and closely related reference sequences**. The numbers above or below the branches correspond to posterior probability (PP) values of the Bayesian analysis. PP values < 50 are not indicated. The scale bar corresponds to the mean number of nucleotide substitutions per site.


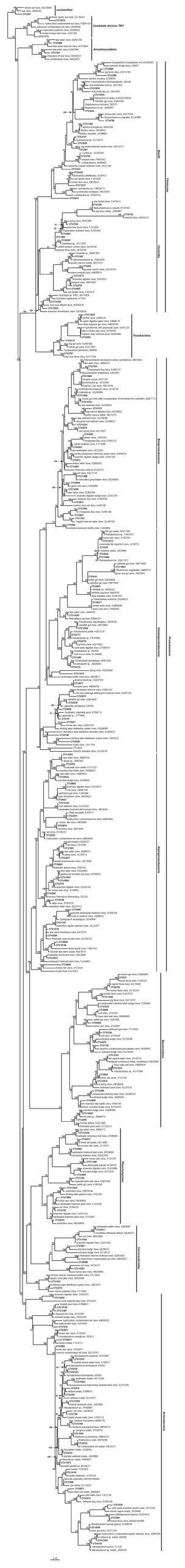


**References of Supplementary table 3**

1. Dubrou, S. *et al.* Diversity, community composition, and dynamics of nonpigmented and late-pigmenting rapidly growing mycobacteria in an urban tap water production and distribution system. *Applied and environmental microbiology* **79,** 5498–508 (2013).

2. Gomila, M., Ramirez, A., Gascó, J. & Lalucat, J. Mycobacterium llatzerense sp. nov., a facultatively autotrophic, hydrogen-oxidizing bacterium isolated from haemodialysis water. *International journal of systematic and evolutionary microbiology* **58,** 2769–73 (2008).

3. Bräuer, S. L. *et al.* Culturable Rhodobacter and Shewanella species are abundant in estuarine turbidity maxima of the Columbia River. *Environmental microbiology* **13,** 589–603 (2011).

4. Glöckner, F. O., Fuchs, B. M., Glo, F. O. & Amann, R. Bacterioplankton Compositions of Lakes and Oceans : a First Comparison Based on Fluorescence In Situ Hybridization Bacterioplankton Compositions of Lakes and Oceans : a First Comparison Based on Fluorescence In Situ Hybridization. (1999).

5. Hiraishi, A., Muramatsu, K. & Urata, K. Characterization of new denitrifying Rhodobacter strains isolated from photosynthetic sludge for wastewater treatment. *Journal of Fermentation and Bioengineering* **79,** 39–44 (1995).

6. Kawasaki, H., Hoshino, Y. & Yamasato, K. Phylogenetic diversity of phototrophic purple non-sulfur bacteria in the Proteobacteria Î± group. *FEMS Microbiology Letters* **112,** 61–66 (1993).

7. Stapleton, R. D., Taylor, D. E. & Sayler, G. S. Quantification of Hyphomicrobium Populations in Activated Sludge from an Industrial Wastewater Treatment System as Determined by 16S rRNA Analysis Quantification of Hyphomicrobium Populations in Activated Sludge from an Industrial Wastewater Treatment Sys. (2000). doi:10.1128/AEM.66.3.1167-1174.2000.Updated

8. Yang, Y., Wang, X., Shi, J. & Li, J. The influence of the discharging sewage on microbial diversity in sludge from Dongting Lake. *World journal of microbiology & biotechnology* **28,** 421–30 (2012).

9. Arenskötter, M., Bröker, D. & Steinbüchel, A. Biology of the metabolically diverse genus Gordonia. *Applied and environmental microbiology* **70,** 3195–204 (2004).

10. Liu, R. *et al.* Diversity of bacteria and mycobacteria in biofilms of two urban drinking water distribution systems. **270,** 261–270 (2012).

11. Wang, C., Zhu, G., Wang, Y., Wang, S. & Yin, C. Nitrous oxide reductase gene (nosZ) and N2O reduction along the littoral gradient of a eutrophic freshwater lake. *Journal of Environmental Sciences (China)* **25,** 44–52 (2013).

12. Ye, L. & Zhang, T. Bacterial communities in different sections of a municipal wastewater treatment plant revealed by 16S rDNA 454 pyrosequencing. *Applied microbiology and biotechnology* **97,** 2681–90 (2013).

13. Zhang, T., Shao, M.-F. & Ye, L. 454 Pyrosequencing Reveals Bacterial Diversity of Activated Sludge From 14 Sewage Treatment Plants. *The ISME journal* **6,** 1137–47 (2012).

14. Boutin, S., Audet, C. & Derome, N. Probiotic treatment by indigenous bacteria decreases mortality without disturbing the natural microbiota of Salvelinus fontinalis. *Canadian journal of microbiology* **59,** 662–70 (2013).

15. Khan, A., Mandal, S., Samanta, D., Chatterjee, S. & Ghosh, K. Phytase-Producing Rhodococcus sp. (MTCC 9508) from Fish Gut: A Preliminary Study. *Proceedings of the Zoological Society* **64,** 29–34 (2011).

16. Kim, D. H., Brunt, J. & Austin, B. Microbial diversity of intestinal contents and mucus in rainbow trout (Oncorhynchus mykiss). *Journal of Applied Microbiology* **102,** 1654–1664 (2007).

17. Sanchez, L. M., Wong, W. R., Riener, R. M., Schulze, C. J. & Linington, R. G. Examining the fish microbiome: vertebrate-derived bacteria as an environmental niche for the discovery of unique marine natural products. *PloS one* **7,** e35398 (2012).

18. Sharifuzzaman, S. M., Al-Harbi, a. H. & Austin, B. Characteristics of growth, digestive system functionality, and stress factors of rainbow trout fed probiotics Kocuria SM1 and Rhodococcus SM2. *Aquaculture* **418-419,** 55–61 (2014).

19. Spanggaard, B. *et al.* The microflora of rainbow trout intestine: a comparison of traditional and molecular identification. *Aquaculture* **182,** 1–15 (2000).

20. Tinh, N. T. N. *et al.* N-acyl homoserine lactone-degrading microbial enrichment cultures isolated from Penaeus vannamei shrimp gut and their probiotic properties in Brachionus plicatilis cultures. *FEMS microbiology ecology* **62,** 45–53 (2007).

21. Holinger, E. P. *et al.* Molecular analysis of point-of-use municipal drinking water microbiology. *Water research* **49,** 225–35 (2014).

22. Liu, R. *et al.* Pyrosequencing analysis of eukaryotic and bacterial communities in faucet biofilms. *The Science of the total environment* **435-436,** 124–31 (2012).

23. Ultee, a., Souvatzi, N., Maniadi, K. & Konig, H. Identification of the culturable and nonculturable bacterial population in ground water of a municipal water supply in Germany. *Journal of Applied Microbiology* **96,** 560–568 (2004).

24. Vílchez, R., Gómez-Silván, C., Purswani, J., González-López, J. & Rodelas, B. Characterization of bacterial communities exposed to Cr(III) and Pb(II) in submerged fixed-bed biofilms for groundwater treatment. *Ecotoxicology (London, England)* **20,** 779–92 (2011).

25. Hahn, M. W. *et al.* Limnohabitans curvus gen. nov., sp. nov., a planktonic bacterium isolated from a freshwater lake. *International journal of systematic and evolutionary microbiology* **60,** 1358–65 (2010).

26. Jezbera, J., Jezberová, J., Kasalický, V., Šimek, K. & Hahn, M. W. Patterns of Limnohabitans microdiversity across a large set of freshwater habitats as revealed by Reverse Line Blot Hybridization. *PloS one* **8,** e58527 (2013).

27. Kasalický, V., Jezbera, J., Simek, K. & Hahn, M. W. Limnohabitans planktonicus sp. nov. and Limnohabitans parvus sp. nov., planktonic betaproteobacteria isolated from a freshwater reservoir, and emended description of the genus Limnohabitans. *International journal of systematic and evolutionary microbiology* **60,** 2710–4 (2010).

28. Šimek, K. *et al.* Differential freshwater flagellate community response to bacterial food quality with a focus on Limnohabitans bacteria. *The ISME journal* **7,** 1519–30 (2013).

29. Zeng, Y., Kasalický, V., Šimek, K. & Koblížeka, M. Genome sequences of two freshwater betaproteobacterial isolates, Limnohabitans species strains Rim28 and Rim47, indicate their capabilities as both photoautotrophs and ammonia oxidizers. *Journal of bacteriology* **194,** 6302–3 (2012).

30. Kasalický, V., Jezbera, J., Hahn, M. W. & Šimek, K. The diversity of the Limnohabitans genus, an important group of freshwater bacterioplankton, by characterization of 35 isolated strains. *PloS one* **8,** e58209 (2013).

31. Besemer, K. *et al.* Unraveling assembly of stream biofilm communities. *The ISME journal* **6,** 1459–68 (2012).

32. Khemkhao, M., Nuntakumjorn, B., Techkarnjanaruk, S. & Phalakornkule, C. Effect of chitosan on UASB treating POME during a transition from mesophilic to thermophilic conditions. *Bioresource technology* **102,** 4674–81 (2011).

33. Kim, Y.-J., Nguyen, N.-L., Weon, H.-Y. & Yang, D.-C. Sediminibacterium ginsengisoli sp. nov., isolated from soil of a ginseng field, and emended descriptions of the genus Sediminibacterium and of Sediminibacterium salmoneum. *International journal of systematic and evolutionary microbiology* **63,** 905–12 (2013).

34. Qu, J. & Yuan, H. Sediminibacterium salmoneum gen. nov., sp. nov., a member of the phylum Bacteroidetes isolated from sediment of a eutrophic reservoir. *International journal of systematic and …* 2191–2194 (2008). doi:10.1099/ijs.0.65514-0

35. Singleton, D. R., Richardson, S. D. & Aitken, M. D. Pyrosequence analysis of bacterial communities in aerobic bioreactors treating polycyclic aromatic hydrocarbon-contaminated soil. *Biodegradation* **22,** 1061–73 (2011).

36. Torrentó, C. *et al.* Enhanced denitrification in groundwater and sediments from a nitrate-contaminated aquifer after addition of pyrite. *Chemical Geology* **287,** 90–101 (2011).

37. Boscaro, V. *et al.* Polynucleobacter necessarius, a model for genome reduction in both free-living and symbiotic bacteria. *Proceedings of the National Academy of Sciences of the United States of America* **110,** 18590–5 (2013).

38. Hahn, M. W. *et al.* The passive yet successful way of planktonic life: genomic and experimental analysis of the ecology of a free-living polynucleobacter population. *PloS one* **7,** e32772 (2012).

39. Vannini, C. *et al.* Endosymbiosis in statu nascendi: close phylogenetic relationship between obligately endosymbiotic and obligately free-living Polynucleobacter strains (Betaproteobacteria). *Environmental microbiology* **9,** 347–59 (2007).

40. Wang, Y., Hammes, F., Boon, N., Chami, M. & Egli, T. Isolation and characterization of low nucleic acid (LNA)-content bacteria. *The ISME journal* **3,** 889–902 (2009).

41. Wu, Q. L. & Hahn, M. W. High predictability of the seasonal dynamics of a species-like Polynucleobacter population in a freshwater lake. *Environmental microbiology* **8,** 1660–6 (2006).

42. Bolnick, D. I. *et al.* Individual diet has sex-dependent effects on vertebrate gut microbiota. *Nature communications* **5,** 4500 (2014).

43. Serkebaeva, Y. M., Kim, Y., Liesack, W. & Dedysh, S. N. Pyrosequencing-based assessment of the bacteria diversity in surface and subsurface peat layers of a northern wetland, with focus on poorly studied phyla and candidate divisions. *PloS one* **8,** e63994 (2013).

44. Smith, D. J. *et al.* Intercontinental dispersal of bacteria and archaea by transpacific winds. *Applied and environmental microbiology* **79,** 1134–9 (2013).

45. Han, S. *et al.* Analysis of bacterial diversity in the intestine of grass carp (Ctenopharyngodon idellus) based on 16S rDNA gene sequences. *Aquaculture Research* **42,** 47–56 (2010).

46. Li, J., Tan, B., Mai, K. & Ai, Q. Responses and Resistance against Vibrio parahaemolyticus Induced by Probiotic Bacterium Arthrobacter XE‐7 in Pacific White Shrimp, Litopenaeus vannamei. *Journal of the World …* **39,** 477–489 (2008).

47. Li, J. *et al.* Comparative study between probiotic bacterium Arthrobacter XE-7 and chloramphenicol on protection of Penaeus chinensis post-larvae from pathogenic vibrios. *Aquaculture* **253,** 140–147 (2006).

48. Merrifield, D. L., Dimitroglou, a, Bradley, G., Baker, R. T. M. & Davies, S. J. Soybean meal alters autochthonous microbial populations, microvilli morphology and compromises intestinal enterocyte integrity of rainbow trout, Oncorhynchus mykiss (Walbaum). *Journal of fish diseases* **32,** 755–66 (2009).

49. Merrifield, D. L., Burnard, D., Bradley, G., Davies, S. J. & Baker, R. T. M. Microbial community diversity associated with the intestinal mucosa of farmed rainbow trout (Oncoryhnchus mykiss Walbaum). *Aquaculture Research* **40,** 1064–1072 (2009).

50. Ringø, E., Sperstad, S., Myklebust, R., Refstie, S. & Krogdahl, Å. Characterisation of the microbiota associated with intestine of Atlantic cod (Gadus morhua L.). *Aquaculture* **261,** 829–841 (2006).

51. Xia, Z., Zhu, M. & Zhang, Y. Effects of the probiotic Arthrobacter sp. CW9 on the survival and immune status of white shrimp (Penaeus vannamei). *Letters in applied microbiology* **58,** 60–4 (2014).

52. Ringø, E., Sperstad, S., Kraugerud, O. F. & Krogdahl, Å. Use of 16S rRNA gene sequencing analysis to characterize culturable intestinal bacteria in Atlantic salmon (Salmo salar) fed diets with cellulose or non-starch polysaccharides from soy. *Aquaculture Research* **39,** 1087–1100 (2008).

53. Cardinali-Rezende, J. *et al.* Organic loading rate and food-to-microorganism ratio shape prokaryotic diversity in a demo-scale up-flow anaerobic sludge blanket reactor treating domestic wastewater. *Antonie van Leeuwenhoek* **104,** 993–1003 (2013).

54. Hartviksen, M. *et al.* Alternative dietary protein sources for Atlantic salmon (Salmo salar L.) effect on intestinal microbiota, intestinal and liver histology and growth. *Aquaculture Nutrition* **20,** 381–398 (2014).

55. Kim, T., Yu, G. H., Jung, J. & Park, H. Bacterial Community Composition and Diversity of a Full-Scale Integrated Fixed-Film Activated Sludge System as Investigated by Pyrosequencing. **20,** 1717–1723 (2010).

56. Piterina, A. V, Bartlett, J. & Pembroke, J. T. Phylogenetic analysis of the bacterial community in a full scale autothermal thermophilic aerobic digester (ATAD) treating mixed domestic wastewater sludge for land spread. *Water research* **46,** 2488–504 (2012).

57. Tsai, Y.-P., You, S.-J., Pai, T.-Y. & Chen, K.-W. Effect of cadmium on composition and diversity of bacterial communities in activated sludges. *International Biodeterioration & Biodegradation* **55,** 285–291 (2005).

58. van Kessel, M. A. *et al.* Pyrosequencing of 16S rRNA gene amplicons to study the microbiota in the gastrointestinal tract of carp (Cyprinus carpio L.). *AMB Express* **1,** 41 (2011).

59. Wu, S., Tian, J., Wang, G., Li, W. & Zou, H. Characterization of bacterial community in the stomach of yellow catfish (Pelteobagrus fulvidraco). *World journal of microbiology & biotechnology* **28,** 2165–74 (2012).

60. Langlois, T. J., Anderson, M. J., Babcock, R. C. & Kato, S. Marine reserves demonstrate trophic interactions across habitats. *Oecologia* **147,** 134–40 (2006).

61. Toivonen, R. K. *et al.* Fermentable fibres condition colon microbiota and promote diabetogenesis in NOD mice. *Diabetologia* **57,** 2183–92 (2014).

62. Adav, S. S., Lee, D.-J. & Lai, J. Y. Microbial community of acetate utilizing denitrifiers in aerobic granules. *Applied microbiology and biotechnology* **85,** 753–62 (2010).

63. Khan, S. T. & Hiraishi, A. Diaphorobacter nitroreducens gen nov, sp nov, a poly(3-hydroxybutyrate)-degrading denitrifying bacterium isolated from activated sludge. *The Journal of general and applied microbiology* **48,** 299–308 (2002).

64. Khardenavis, A. a, Kapley, A. & Purohit, H. J. Simultaneous nitrification and denitrification by diverse Diaphorobacter sp. *Applied microbiology and biotechnology* **77,** 403–9 (2007).

65. Sadaie, T. *et al.* Reducing Sludge Production and the Domination of Comamonadaceae by Reducing the Oxygen Supply in the Wastewater Treatment Procedure of a Food-Processing Factory. *Bioscience, Biotechnology, and Biochemistry* **71,** 791–799 (2007).

66. Khan, S. T., Horiba, Y., Yamamoto, M. & Hiraishi, A. Members of the Family Comamonadaceae as Primary Poly(3-Hydroxybutyrate-co-3-Hydroxyvalerate)-Degrading Denitrifiers in Activated Sludge as Revealed by a Polyphasic Approach. *Applied and environmental microbiology* **68,** 3206–3214 (2002).

67. Tabrez Khan, S. & Hiraishi, a. Isolation and characterization of a new poly(3-hydroxybutyrate)-degrading, denitrifying bacterium from activated sludge. *FEMS microbiology letters* **205,** 253–7 (2001).

68. Takahashi, M., Yamada, T., Tanno, M., Tsuji, H. & Hiraishi, A. Nitrate Removal Efficiency and Bacterial Community Dynamics in Denitrification Processes Using Poly (L-lactic acid) as the Solid Substrate. *Microbes and Environments* **26,** 212–219 (2011).

69. Weissbrodt, D. G., Schneiter, G. S., Fürbringer, J.-M. & Holliger, C. Identification of trigger factors selecting for polyphosphate- and glycogen-accumulating organisms in aerobic granular sludge sequencing batch reactors. *Water research* **47,** 7006–18 (2013).

70. Madigan, M., Cox, S. S. & Stegeman, R. a. Nitrogen fixation and nitrogenase activities in members of the family Rhodospirillaceae. *Journal of bacteriology* **157,** 73–8 (1984).

71. Road, S. P. Anaerobic respiration in the Rhodospirillaceae " characterisation of pathways and evaluation of roles in redox balancing during photosynthesis. **46,** 117–143 (1987).

72. Sheu, S.-Y., Chen, Y.-L., Young, C.-C. & Chen, W.-M. Lacibacterium aquatile gen. nov., sp. nov., a new member of the family Rhodospirillaceae isolated from a freshwater lake. *International journal of systematic and evolutionary microbiology* **63,** 4797–804 (2013).

73. Adav, S. S., Lee, D.-J. & Lai, J.-Y. Potential cause of aerobic granular sludge breakdown at high organic loading rates. *Applied microbiology and biotechnology* **85,** 1601–10 (2010).

74. Ebrahimi, S. *et al.* Performance and microbial community composition dynamics of aerobic granular sludge from sequencing batch bubble column reactors operated at 20 degrees C, 30 degrees C, and 35 degrees C. *Applied microbiology and biotechnology* **87,** 1555–68 (2010).

75. Fernández, N., Sierra-Alvarez, R., Amils, R., Field, J. a & Sanz, J. L. Compared microbiology of granular sludge under autotrophic, mixotrophic and heterotrophic denitrification conditions. *Water science and technology* **59,** 1227–36 (2009).

76. Guo, F., Zhang, S.-H., Yu, X. & Wei, B. Variations of both bacterial community and extracellular polymers: the inducements of increase of cell hydrophobicity from biofloc to aerobic granule sludge. *Bioresource technology* **102,** 6421–8 (2011).

77. Quan, Z.-X., Im, W.-T. & Lee, S.-T. Azonexus caeni sp. nov., a denitrifying bacterium isolated from sludge of a wastewater treatment plant. *International journal of systematic and evolutionary microbiology* **56,** 1043–6 (2006).

78. Arias, C. R., Koenders, K. & Larsen, A. M. Predominant bacteria associated with red snapper from the Northern Gulf of Mexico. *Journal of aquatic animal health* **25,** 281–9 (2013).

79. Caipang, C. M. A., Brinchmann, M. F. & Kiron, V. Antagonistic activity of bacterial isolates from intestinal microbiota of Atlantic cod, Gadus morhua , and an investigation of their immunomodulatory capabilities. *Aquaculture Research* **41,** 249–256 (2010).

80. Gram, L., Melchiorsen, J. & Bruhn, J. B. Antibacterial activity of marine culturable bacteria collected from a global sampling of ocean surface waters and surface swabs of marine organisms. *Marine biotechnology (New York, N.Y.)* **12,** 439–51 (2010).

81. Meziti, A., Mente, E. & Kormas, K. A. Gut bacteria associated with different diets in reared Nephrops norvegicus. *Systematic and applied microbiology* **35,** 473–82 (2012).

82. Ray, a. K., Ghosh, K. & Ringø, E. Enzyme-producing bacteria isolated from fish gut: a review. *Aquaculture Nutrition* **18,** 465–492 (2012).

83. Smriga, S., Sandin, S. a & Azam, F. Abundance, diversity, and activity of microbial assemblages associated with coral reef fish guts and feces. *FEMS microbiology ecology* **73,** 31–42 (2010).

84. Sugita, H., Mizuki, H. & Itoi, S. Diversity of siderophore-producing bacteria isolated from the intestinal tracts of fish along the Japanese coast. *Aquaculture Research* **43,** 481–488 (2012).

85. Svanevik, C. S. & Lunestad, B. T. Characterisation of the microbiota of Atlantic mackerel (Scomber scombrus). *International journal of food microbiology* **151,** 164–70 (2011).

86. Fernandez-Piquer, J., Bowman, J. P., Ross, T. & Tamplin, M. L. Molecular analysis of the bacterial communities in the live Pacific oyster (Crassostrea gigas) and the influence of postharvest temperature on its structure. *Journal of applied microbiology* **112,** 1134–43 (2012).

87. Finnegan, L. *et al.* A survey of culturable aerobic and anaerobic marine bacteria in de novo biofilm formation on natural substrates in St. Andrews Bay, Scotland. *Antonie van Leeuwenhoek* **100,** 399–404 (2011).

88. Graue, J., Engelen, B. & Cypionka, H. Degradation of cyanobacterial biomass in anoxic tidal-flat sediments: a microcosm study of metabolic processes and community changes. *The ISME journal* **6,** 660–9 (2012).

89. Li, S. *et al.* The intestinal microbial diversity in mud crab (Scylla paramamosain) as determined by PCR-DGGE and clone library analysis. *Journal of applied microbiology* **113,** 1341–51 (2012).

90. Madigan, T. L. *et al.* A microbial spoilage profile of half shell Pacific oysters (Crassostrea gigas) and Sydney rock oysters (Saccostrea glomerata). *Food microbiology* **38,** 219–27 (2014).

91. Zhao, J.-S., Manno, D. & Hawari, J. Psychrilyobacter atlanticus gen. nov., sp. nov., a marine member of the phylum Fusobacteria that produces H2 and degrades nitramine explosives under low temperature conditions. *International journal of systematic and evolutionary microbiology* **59,** 491–7 (2009).

92. Amato, L. *et al.* Microbial composition of defect smear - A problem evolving during foil-prepacked storage of red-smear cheeses. *International Dairy Journal* **27,** 77–85 (2012).

93. Laclaire, L. L. & Facklam, R. R. Comparison of three commercial rapid identification systems for the unusual gram-positive cocci Dolosigranulum pigrum, Ignavigranum ruoffiae, and Facklamia species. *Journal of Clinical Microbiology* **38,** 2037–2042 (2000).

94. Pérez Alonso, a. J. J., Husein-El Ahmed, H., Del Olmo Rivas, C., Caballero Marcos, L. & Pérez Ramon, J. a. A. Facklamia sourekii necrotizing gangrene. *Médecine et Maladies Infectieuses* **42,** 283–284 (2012).

95. Rasolofo, E. A., St-Gelais, D., LaPointe, G. & Roy, D. Molecular analysis of bacterial population structure and dynamics during cold storage of untreated and treated milk. *International Journal of Food Microbiology* **138,** 108–118 (2010).

96. Roth, E., Schwenninger, S. M., Eugster-Meier, E. & Lacroix, C. Facultative anaerobic halophilic and alkaliphilic bacteria isolated from a natural smear ecosystem inhibit Listeria growth in early ripening stages. *International Journal of Food Microbiology* **147,** 26–32 (2011).

97. Takamatsu, D., Ide, H., Osaki, M. & Sekizaki, T. Identification of Facklamia sourekii from a lactating cow. *The Journal of veterinary medical science / the Japanese Society of Veterinary Science* **68,** 1225–1227 (2006).

98. Wang, B. *et al.* Molecular analysis of the relationship between specific vaginal bacteria and bacterial vaginosis metronidazole therapy failure. *European Journal of Clinical Microbiology & Infectious Diseases* **33,** 1749–56 (2014).

99. Hahn, M. W., Stadler, P., Wu, Q. L. & Pöckl, M. The filtration-acclimatization method for isolation of an important fraction of the not readily cultivable bacteria. *Journal of microbiological methods* **57,** 379–90 (2004).

100. Sethi, S., Datta, A., Gupta, B. L. & Gupta, S. Optimization of Cellulase Production from Bacteria Isolated from Soil. *ISRN Biotechnology* **2013,** 1–7 (2013).

101. Boutin, S., Bernatchez, L., Audet, C. & Derôme, N. Network analysis highlights complex interactions between pathogen, host and commensal microbiota. *PloS one* **8,** e84772 (2013).

102. McDonald, R., Schreier, H. J. & Watts, J. E. M. Phylogenetic analysis of microbial communities in different regions of the gastrointestinal tract in Panaque nigrolineatus, a wood-eating fish. *PloS one* **7,** e48018 (2012).

103. Askarian, F., Zhou, Z., Olsen, R. E., Sperstad, S. & Ringø, E. Culturable autochthonous gut bacteria in Atlantic salmon (Salmo salar L.) fed diets with or without chitin. Characterization by 16S rRNA gene sequencing, ability to produce enzymes and in vitro growth inhibition of four fish pathogens. *Aquaculture* **326-329,** 1–8 (2012).

104. Feng, J.-B., Hu, C.-Q., Luo, P., Zhang, L.-P. & Chen, C. Microbiota of yellow grouper (Epinephelus awoora Temminck & Schlegel, 1842) fed two different diets. *Aquaculture Research* **41,** 1778–1790 (2010).

105. Paungfoo, C., Prasertsan, P., Burrell, P. C., Intrasungkha, N. & Blackall, L. L. Nitrifying Bacterial Communities in an Aquaculture Wastewater Treatment System Using Fluorescence In Situ Hybridization ( FISH ), 16S rRNA Gene Cloning , and Phylogenetic Analysis. **97,** 985–990 (2007).

106. Sugita, H., Nakamura, H. & Shimada, T. Microbial communities associated with filter materials in recirculating aquaculture systems of freshwater fish. *Aquaculture* **243,** 403–409 (2005).

107. Kruse, M. *et al.* Relevance and diversity of Nitrospira populations in biofilters of brackish RAS. *PloS one* **8,** e64737 (2013).

108. Schreier, H. J., Mirzoyan, N. & Saito, K. Microbial diversity of biological filters in recirculating aquaculture systems. *Current opinion in biotechnology* **21,** 318–25 (2010).

109. Itoi, S., Ebihara, N., Washio, S. & Sugita, H. Nitrite-oxidizing bacteria, Nitrospira, distribution in the outer layer of the biofilm from filter materials of a recirculating water system for the goldfish Carassius auratus. *Aquaculture* **264,** 297–308 (2007).

110. Brown, M. N., Briones, A., Diana, J. & Raskin, L. Ammonia-oxidizing archaea and nitrite-oxidizing nitrospiras in the biofilter of a shrimp recirculating aquaculture system. *FEMS microbiology ecology* **83,** 17–25 (2013).

111. Mußmann, M. *et al.* Colonization of freshwater biofilms by nitrifying bacteria from activated sludge. *FEMS microbiology ecology* **85,** 104–15 (2013).

112. Hovanec, T. A., Taylor, L. T., Blakis, A. & Delong, F. Nitrospira -Like Bacteria Associated with Nitrite Oxidation in Freshwater Aquaria Nitrospira -Like Bacteria Associated with Nitrite Oxidation in Freshwater Aquaria. **64,** (1998).

113. Cébron, A., Berthe, T. & Garnier, J. Nitrification and Nitrifying Bacteria in the Lower Seine River and Estuary ( France ) Nitrification and Nitrifying Bacteria in the Lower Seine River and Estuary ( France ). **69,** (2003).

114. Tamaki, H. *et al.* Comparative Analysis of Bacterial Diversity in Freshwater Sediment of a Shallow Eutrophic Lake by Molecular and Improved Cultivation-Based Techniques Comparative Analysis of Bacterial Diversity in Freshwater Sediment of a Shallow Eutrophic Lake by Molecul. (2005). doi:10.1128/AEM.71.4.2162

115. Altmann, D., Stief, P., Amann, R., Beer, D. De & Schramm, A. Brief report In situ distribution and activity of nitrifying bacteria in freshwater sediment. **5,** 798–803 (2003).

116. Dulger, S., Demirbag, Z. & Belduz, A. O. Anoxybacillus ayderensis sp. nov. and Anoxybacillus kestanbolensis sp. nov. *International journal of systematic and evolutionary microbiology* **54,** 1499–503 (2004).

117. Namsaraev, Z. B. *et al.* Anoxybacillus mongoliensis sp. nov., a novel thermophilic proteinase producing bacterium isolated from alkaline hot spring, Central Mongolia. *Microbiology* **79,** 491–499 (2010).

118. Canakci, S., Kacagan, M., Inan, K., Belduz, A. O. & Saha, B. C. Cloning, purification, and characterization of a thermostable alpha-L-arabinofuranosidase from Anoxybacillus kestanbolensis AC26Sari. *Applied microbiology and biotechnology* **81,** 61–8 (2008).

119. Yassin, a. F., Kroppenstedt, R. M. & Ludwig, W. Corynebacterium glaucum sp. nov. *International Journal of Systematic and Evolutionary Microbiology* **53,** 705–709 (2003).

120. Wendisch, V. F., De Graaf, A. a., Sahm, H. & Eikmanns, B. J. Quantitative determination of metabolic fluxes during coutilization of two carbon sources: Comparative analyses with Corynebacterium glutamicum during growth on acetate and/or glucose. *Journal of Bacteriology* **182,** 3088–3096 (2000).

121. Inui, M. *et al.* Metabolic analysis of Corynebacterium glutamicum during lactate and succinate productions under oxygen deprivation conditions. *Journal of Molecular Microbiology and Biotechnology* **7,** 182–196 (2004).
